# Supporting respiratory epithelia and lowering inflammation to effectively treat common cold symptoms: A randomized controlled trial

**DOI:** 10.1371/journal.pone.0301959

**Published:** 2024-11-27

**Authors:** Pavel Pugach, Nazlie Sadeghi-Latefi

**Affiliations:** Applied Biological Laboratories Inc, New York, NY, United States of America; PearResearch / Government Doon Medical College, INDIA

## Abstract

Common cold viruses are leading triggers of asthma attacks, causing nearly two million hospitalizations per year and productivity losses approaching $40B. They also increase susceptibility to bacterial infections driving antibiotic use. Post-market clinical studies have questioned the efficacy of most over the counter (OTC) cough and cold ingredients against placebo in treating various symptoms. To our knowledge, only aspirin significantly improved overall illness severity compared to placebo and that was by about 25–30%. In this double-blind randomized placebo-controlled trial involving 157 participants, we sought to determine whether a throat spray containing a mucosal immune complex (MIC) (comprised of lysozyme, lactoferrin, and aloe) along with anti-inflammatory salicylates can increase the hereto reported efficacy of aspirin at reducing common cold symptoms. Previously published reports showed that the MIC can protect respiratory epithelia and lower inflammatory cytokines. Salicylates are naturally occurring plant compounds found in many common foods as well as wintergreen oil and are chemically similar precursors to aspirin (acetyl salicylate). Participants self-administered treatments (throat sprays every hour and tablets every four hours) and completed surveys at home over two days. Treatments included MIC spray mixed with 6 mg aspirin + placebo tablet (Treatment 1), MIC spray mixed with 6 mg wintergreen oil+ placebo tablet (Treatment 2), MIC spray mixed with 6mg wintergreen oil+ 325 mg aspirin tablet (Treatment 3). Participants included adult volunteers ages 21–66 (average 44), 54% female, 46% male, 46% African American, 8% Asian, 39% Caucasian, and 7% Hispanic, having common cold symptoms lasting less than two days. The main outcome measures included Sore Throat Pain Intensity (STPIS) 0–100 at 36 hours (primary endpoint) and Modified Jackson Score (MJS), a combination of eight cold symptoms (secondary endpoint). Both primary and secondary endpoints were met. Sore throat pain as measured by STPIS decreased 68–75% by 36 hours depending on treatment. Other symptoms such as nasal discharge, congestion, sneezing, cough, sore throat, and malaise as measured by MJS decreased 38–68% depending on treatment. In repeated measure within group analysis observing the same participants over multiple time points; the mean change of STPIS values and their percentage change from baseline to 36 hours were as follows: Placebo (-7.84 [95% CI -14.20 to -1.47];(-14%)), Treatment 1 (-42.41 [95% CI -48.30 to -36.52];(-75%)), Treatment 2 (-38.60 [95% CI -46.64 to -31.56];(-68%)), and Treatment 3 (-44.19 [95% CI -52.11 to -36.27];(-79%)). In repeated measure within group analysis all treatments significantly reduced cold symptom severity (MJS) from Days 1–2. Results were as follows: Treatment 1 (-2.26 [95% CI -3.04 to -1.47] (-38%)), Treatment 2 (-3.81 [95% CI -4.82 to -2.80];(-53%)), Treatment 3 (-4.49 [95% CI -5.62 to -3.57];(-69%)). As a result of this study, we conclude that supporting upper respiratory epithelia and reducing COX-mediated inflammation may be used to effectively treat common cold symptoms.

**Trial registration:** ClinicalTrials.gov Identifier: NCT06106880 Posted 30/10/2023.

## Introduction

The common cold is a symptom-based disease caused by regularly circulating respiratory viruses excluding influenza and SARS or MERS associated coronaviruses [1]. Its economic impact is estimated at $40 billion in the US [2,3]. Despite the public health burden, there are no clinically proven, Food and Drug Administration (FDA) approved drugs, or other remedies to effectively lower symptom severity or shorten duration of illness [1].

Over the counter (OTC) drugs for common cold symptoms contain ingredients allowed by the FDA under OTC Monographs and their administrative orders as of 1972. Since then, post-market clinical studies have questioned the efficacy of many of these ingredients against placebo in treating various symptoms. Efficacy of dextromethorphan [4–8], guaifenesin [9,10], pseudoephedrine [11], and benzocaine [12,13] have all been questioned. The efficacy of pseudoephedrine for the treatment of nasal congestion is questionable [14,15], and it has been shown to exacerbate conditions such as hypertension and restless leg syndrome [11,16]. Its role as a key ingredient in the formulation of illicit substances led to its behind-the-counter regulation [17] and has subsequently been replaced by phenylephrine in OTC cold products over the last 15 years. Several studies found that phenylephrine is not different from placebo in treating cold symptoms [18–22]. In September 2023, as FDA panel issued the ruling that oral phenylephrine, grossing over $1.5 billion in the last year alone, is not effective for the treatment of cold and flu symptoms [23]. Ibuprofen, and acetaminophen effectively improve mainly fever and pain symptoms [24,25]; but to our knowledge, not any validated measure of overall illness. Furthermore, according to some studies, prenatal use of acetaminophen has been associated with a 19% and 21% increase in the risk of autism spectrum disorder and attention deficit disorder, respectively [26]. In addition, therapeutic doses of acetaminophen have been shown to alter liver function, as well as significantly deplete glutathione, an important endogenous antioxidant [27–30]. To our knowledge, only aspirin (with vitamin C) significantly improved overall illness severity as measured by the Wisconsin Upper Respiratory Symptom Score by about 25–30% [31]. Aspirin is a well-known irreversible COX-enzyme inhibitor with anti-inflammatory effects. COX enzymes have been shown in numerous studies to induce prostaglandin formation which leads to common cold symptoms [32–36]. Upon sensing injury at the respiratory epithelium, bradykinin induces release of arachidonic acid (AA) from cell membranes via phospholipase A2, and AA is then converted to prostaglandin E2 via COX enzymes [37]. Methyl salicylate is a major constituent of wintergreen oil and a COX-enzyme inhibitor analogous to aspirin. Repairing the epithelia and controlling inflammation are critical to limiting symptoms [38].

We treated people exhibiting naturally acquired common cold symptoms with a throat spray containing a Mucosal Immune Complex (MIC) and various combinations of aspirin, wintergreen oil, and menthol. The aspirin was either mixed into the throat spray (6 mg) or taken as a tablet (325mg). The MIC contained lysozyme, lactoferrin and aloe, natural dietary supplements which lubricate and protect the respiratory barrier [39] and which may also affect rheological properties of the mucosal surface [40] or act as non-specific glycoprotein attachment inhibitors [41]. Lactoferrin binds to multiple viruses, blocking their entry into epithelial cells, induces type I interferon production and enhances Th1 responses in the context of viral infection [42–46]. Lactoferrin also prevents and repairs the virus-induced cytotoxicity in host cells, thereby limiting the release of damage-induced pro-inflammatory cytokines that correlate with symptoms [46]. Lysozyme has antimicrobial effects and may work in synergy with lactoferrin through unknown mechanisms [47]. Reduced levels of lysozyme and lactoferrin in the mucosa increases susceptibility to infections and leads to more severe illness, further supporting their role in mucosal health [48,49]. According to previously published studies using human respiratory organoid tissues, the MIC augmented aspirin’s anti-inflammatory effects possibly by protecting or buffering the respiratory epithelia [39].

## Methods

The protocol for this randomized, placebo-controlled clinical trial was approved by the Advarra Institutional Review Board and written informed consent was obtained for all participants. This study followed the Consolidated Standards of Reporting Trials (CONSORT) reporting guidelines. The protocol for this active-comparator, parallel-arm RCT (NCT06106880) has been published on clinicaltrials.gov.

### Trial participants

The trial was a 13-month multi-center randomized clinical trial conducted from May 2022 to June 2023 in participants’ homes in Washington DC, Baltimore MD, New York NY, Atlanta, GA, Houston TX, and Orange County CA. Inclusion criteria were: healthy adults aged 18–65, experiencing a sore throat rated at least 3 on a 10-point scale, and a sore throat duration of less than 48 hours at the time of intake assessment. Exclusion criteria included: sore throat exceeding two full days, likelihood of strep throat, allergies to eggs, milk, or aspirin, pregnancy, presence of chronic disease, recent history of allergy, fever above 101°F, ACE inhibitor use, participation in another clinical trial, and smoking.

Subjects were recruited through targeted social media advertisements. Interested individuals contacted the Clinical Trial Manager (CTM) who conducted a phone interview using a standardized script. Responses were recorded in a digital form (CTM Intake Form) on a HIPAA-compliant Survey Monkey^TM^ platform. Participants deemed eligible were randomized, seen at home by the Clinical Trial Administrator (CTA), consented, and given blind coded kits containing treatment or placebo. Prior to commencing treatment, the following morning, participants met with the PI by video conference. Personally identifiable information was securely stored on password-protected mobile devices and databases, accessible only to authorized personnel.

### Randomization and blinding

180 eligible participants were randomized 1:1:1:1 to receive one of three treatments or placebo. The baseline characteristics of the study participants in each group were similar and are shown in Table 1. Kits containing treatments or placebo, instructions, and survey questionnaires were provided to patients at home after signing informed consent. Randomization was performed by a third-party Clinical Research Organization (CRO) who manufactured and bottled the treatments and placebo. Codes were generated by a computer program using the rand function which uses the Mersenne Twister algorithm [50]. A total of 400 codes were assigned, with each code representing one kit, then four treatment groups were established for participant allocation. Random codes were generated in blocks of 8 to maintain balanced group sizes after every 8 patients were randomized. The code format consisted of a two-digit investigator number followed by a four-digit subject identifier (e.g., XX-XXXX), enabling unique identification of each participant and their assigned kit. Each participant received the treatment they were originally randomized to receive. Throughout the study and until statistical analysis was complete only the CRO and the physician on call for adverse events, the Adverse Events Specialist (AES) was informed which kit numbers were assigned to which treatment groups.

**Table 1:** Demographics of randomized participants across treatment groups.

Variable	Treatment Group 1 (n = 43)	Treatment Group 2 (n = 42)	Treatment Group 3 (n = 37)	Placebo Group (n = 35)
Mean Age (SD)	47.0 (13.2)	46.6 (14.2)	41.2 (12.4)	44.2 (13.5)
Body-Mass Index (SD)	27.7 (6.9)	28.4 (8.8)	28.6 (6.2)	26.3 (5.2)
Male Gender (%)	49	50	45	40
Mean Intake Sore Throat Rating (0–10) (SD)	6.7 (3.9)	6.0 (1.6)	6.3 (1.7)	5.9 (1.6)
Sore Throat Duration at Intake (hours) (SD)	38.0 (12.0)	39.4 (11.6)	36.6 (12.1)	39.8 (11.6)
Mean body temperature (°F)	95.6	97.5	97.3	96.3
Caucasian (%)	42	36	37	37
African American (%)	49	45	47	47
Hispanic/Latinx (%)	5	10	5	5
Asian (%)	5	10	11	11
Atlanta, GA site (%)*	33	36	45	34

* Of 6 US sites, Atlanta GA had the greatest enrollment followed by New York City at 27.7%.

### Intervention groups

All three treatment groups contained the mucosal immune complex (MIC) which was composed of 0.5% bovine lactoferrin (Ingredia, Arras, France), 5% chicken egg white lysozyme (NutriScience Innovation, Milford, CT) and 0.2% whole leaf aloe vera juice (365 Brand Austin, TX) in a buffered aqueous solution (pH 6.5). The buffered aqueous solution was administered from one side of a two headed spray bottle. The second side contained an organic solvent comprised of propylene glycol, ethyl alcohol, and glycerin in which menthol, aspirin (Perrigo, Chicago, IL), and/or wintergreen oil (Now Foods, Bloomindale, IL) were dissolved. The individual treatment groups included: MIC spray with 0.6% aspirin (6 mg per spray dose) + placebo tablet (Treatment 1); MIC spray with 0.6% wintergreen oil (6 mg per spray dose) + placebo tablet (Treatment 2); and MIC spray with 0.6% wintergreen oil + 325 mg aspirin tablet (Treatment 3). Wintergreen was derived from Gaultheria procumbens (the American wintergreen plant) and is comprised of 98% methylsalicylate. Sprays for Treatments 1–3 contained menthol at a concentration of 0.5% (5 mg per spray dose). The placebo spray contained 0.0009% (0.009 mg per spray dose) menthol, in the same organic buffer used in the treatment groups, a sub-therapeutic dose for taste. The aqueous phase of the placebo spray contained the same buffers as the treatment groups but did not contain MIC. The treatment throat sprays and aspirin pills were identical in taste and appearance to placebo throat sprays and placebo pills.

Sprays were administered each waking hour for two days. Sprays delivered 0.5 mL per actuation from each side of a two-sided spray bottle for a total of 1 ml per dose. One set of two sprays was recommended per waking hour for a total of 12 mL per day or 72 mg of aspirin. The pills were taken every 4 hours. A daily dose of 1.372 g of aspirin was administered, which falls within the recommended adult daily dose of the US Food and Drug Administration.

Once enrolled, per the protocol, participants were seen via telemedicine by the Principal Investigator (PI). The PI directed them to begin treatment as well as a checklist of daily surveys on the morning of the following day. Immediately before their first treatment and at 4-hour intervals during waking hours (3 times per day), participants recorded their pain levels on the STPIS Visual Analog Scale (VAS). A Jackson score questionnaire was completed at the end of each day to assess symptom intensity. Once the two days were completed, the participants mailed their forms to the PI. The PI and CTM were available to address non-urgent matters, while the Adverse Events Specialist (AES) handled serious adverse events and unblinded a participant if necessary.

### Outcomes measures

The primary outcome was difference in pain intensity on the STPIS over a 36-hour period following the administration of the first dose of medication, as measured by the STPIS VAS on a 100mm scale. The application of visual analog scales for assessing sore throat pain, as well as various other types of pain, has been widely validated through extensive research studies [51]. All STPIS lines on the paper patient forms were 100mm in length with “no pain” on the left side and “severe pain” on the right side of the line. Participants were instructed to physically mark the place on the line that corresponded to the pain they felt in their throat. The patient forms were then digitally scanned by Econometrica Inc Research and Management, Bethesda, Maryland, the data management company (DMC) as they were received. To convert the marks to numeric values, the DMC used the Adobe measuring tool to measure the length of the entire line and normalize for minor changes that may have resulted from the scanning process. This was calculated as the denominator. The numerator was then calculated as the distance from the left side of the line to the mark (or the center of the mark if the mark was not perpendicular). The division of the numerator by the denominator then gave the STPIS value which was converted to a 100 point scale (multiplied by 100) to generate the final STPIS value.

Considering the importance of overall illness severity, the secondary outcome was reduction in cold symptoms as measured by changes in the Modified Jackson Score by the second day. The Jackson Score is a clinically validated measure of overall illness severity, expressed as a combination of eight major common cold symptoms including sneezing, coughing, fever and chills [52,53]. To perform quantitative analysis of Jackson Scores, the categorical responses were recoded to numeric values, based on the following formula: Absent = 0, Mild = 1, Moderate = 2, Severe = 3. Next, the Modified Jackson Score was calculated as the sum of the numeric values from all symptom assessments. The total symptom score could range from 0–24 with higher numbers representing higher symptom burden.

### Data acquisition

Participants mailed their survey forms to a secure PO Box accessible only to the PI. Forms did not contain any personal information, only the randomized kit number assigned to that participant. The PI did not share the data with anyone besides the DMC to which they were delivered and scanned for blinded analysis.

### Statistical analysis

All statistical analyses were carried out blind by the DMC. Treatment groups were unmasked by the AES when the data analysis was complete. The primary endpoint was change in STPIS from treatment initiation to 36 hours (Day 1, 1^st^ entry to Day 2, 4^th^ entry). The secondary endpoint was change in Modified Jackson Score (MJS) from Day 1 to Day 2. The mean, 95% confidence interval (CI) of the mean, standard deviation, and median were calculated for each measurement (for both days) to demonstrate the basic characteristics of the data.

To analyze the changes in scores over time and assess the impact of the interventions, repeated-measures analysis was implemented as a multivariate ANOVA. In addition, post-hoc analysis–pairwise group comparisons–was conducted to identify specific differences between each group pair after finding a statistically significant overall impact. Tukey-Kramer adjustments were used for multiple comparisons.

### Sample size justification and interim analysis

A previous study utilizing an STPIS endpoint [50] following administration of flurbiprofen (a non-steroidal anti-inflammatory drug (NSAID) reported a 59% decrease (-196.6 mm x h [95% CI -321 to -72.2]; p<0.01) using as its endpoint a time weighted sum difference over 24 hours. A total of 198 subjects was the sample size for this two-group RCT with 1:1 randomization that compared placebo to treatment [51]. This same sample size was adopted for our study.

We conducted a double-blind placebo controlled RCT with one placebo arm and three active treatment arms using various doses and 1:1:1:1 randomization. Prompted by an ethical concern that pain scores from one of the groups were markedly higher than the others (and since trial participants could not take other medication), the independent DMC conducted an interim analysis based on 159 subjects. The analysis was conducted at the end of the 2023 cold and flu season, after the study had been ongoing for one year. All statistical calculations were performed by the DMC while blind. When unblinded, the group with the higher pain scores was discovered to be the placebo group and efficacy of all three active treatment groups far exceeded expectations. A highly statistically significant difference was seen among all treatment groups when the primary endpoint was examined (p < 0.0001). The three active treatments showed on average a 70% improvement in the primary efficacy endpoint relative to placebo. Conditional power analyses were conducted and the probability that the trial results would be non-significant at the planned end of the study was close to zero. Analyses of secondary endpoints as well as between-group comparisons were performed and reviewed by the DMC before the treatment assignments were unblinded. Analysis of secondary endpoints showed similar levels of improvement relative to placebo and were supportive of the primary hypothesis. Therefore, with 40% of the projected sample size accrued and analyzed, the decision was made to terminate the study based on 159 subjects (approximately 40 subjects per group).

## Results

### Participants, retention, and fidelity

**Fig 1:**
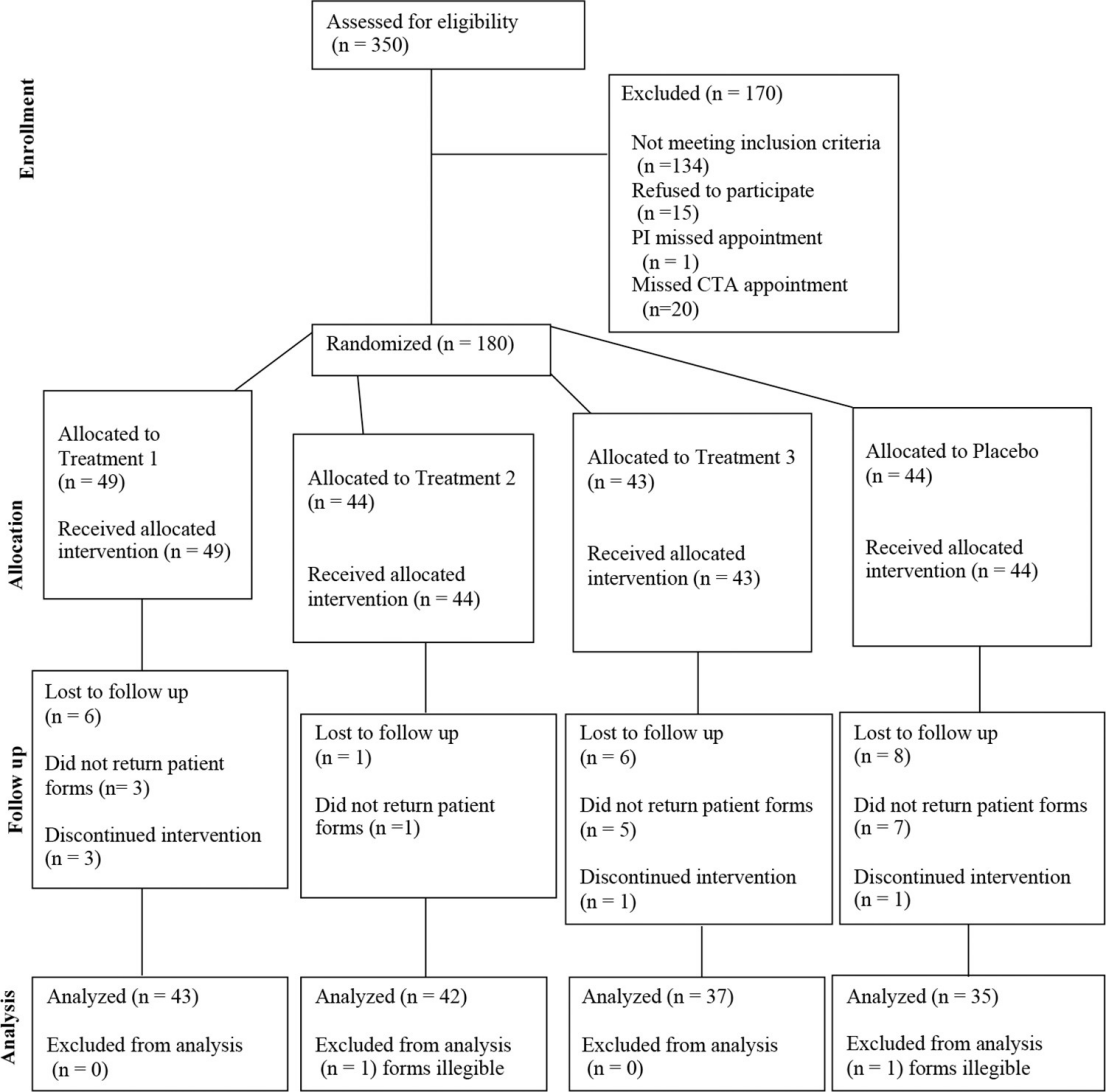
Participant flow diagram. CONSORT diagram showing the number of participants who were assessed for eligibility, excluded, randomized, and lost to follow up through the course of the study.

Of 350 individuals initially screened by phone (by the CTM), 180 were subsequently screened in person (by the CTA), consented, and randomized (Fig 1). Following randomization, 21 individuals were lost to follow up: 16 did not mail in their forms and 5 were discontinued by the PI. Of those discontinued by the PI, 1 individual missed their appointment, 1 was discontinued for an adverse event, and 3 were deemed ineligible. Two participants returned illegible forms and were excluded from analysis by the DMC before unblinding. Baseline characteristics of randomized individuals were similar between groups as shown in Table 1. The number of participants lost to follow up due to not returning forms, was lower for Treatment 2 than the other groups, but similar for Placebo and Treatments 1 and 3. (Fig 1). No differences in baseline characteristics were observed between participants lost to follow up across the four groups, or compared to those who completed the study, therefore we did not record any observable attrition bias. All study participants lost to follow-up were excluded from analysis. Of those randomized, 44 were allocated to placebo, 49 to Treatment 1, 44 to Treatment 2, and 43 to Treatment 3. All participants were analyzed in their randomized group, and there was no between arm cross-over. In the final analyses there were 35 in the placebo group, 43 in Treatment 1, 42 in Treatment 2, and 37 in Treatment 3 due to loss to follow up as explained above and as seen in Fig 1. There were no reported issues with safety and tolerability in any of the treatment groups. One adverse event occurred, but deemed unrelated to treatment by the AES. The participant was nevertheless asked to stop participating in the study by the AES.

Per Table 1, the 180 randomized participants had baseline mean (SD) age of 43.5(13.5) years, and BMI of 27.4 (6.2). A total of 96 (52.5%) participants were women; 79 (43.9%) were African American, 18 (10%) were Hispanic/Latinx, 71 (39.4%) were Caucasian, and 12 (6.7%) were Asian. The attrition rate (participants lost to follow up) was 12.8% which included the 23 participants lost to follow up.

All tables and graphic representations of data were prepared by the DMC.

### Primary outcome

We used STPIS to evaluate the Treatment effects on sore throat pain over time. Effects of STPIS over time were analyzed using repeated measures analysis to measure within subject changes. The mean, 95% confidence interval (CI) of the mean, and median for STPIS measures (Tables 2 and 3) summarize the distribution and central tendency of the data. STPIS analyses were obtained four times per day over two days for each participant.

**Table 2:** Descriptive statistics for STPIS measurements on Day 1. Survey data for each participant was collected upon waking and every four hours thereafter during waking hours.

		**Day 1 1st Entry**	**Day 1 2nd Entry**	**Day 1 3rd Entry**	**Day 1 4th Entry**
Group	Count (Percent)	Mean (95% CI)	Mean (95% CI)	Mean (95% CI)	Mean (95% CI)
Placebo	35 (22.3%)	55.4 (47.6–63.1)	54.5 (46.3–62.7)	50.5 (42.9–58.2)	44.6 (36.3–53.0)
Treatment 1	43 (27.4%)	56.8 (49.9–63.7)	46.9 (39.8–54.1)	39.8 (33.0–46.5)	33.8 (27.2–40.5)
Treatment 2	42 (26.8%)	56.6 (49.7–63.6)	49.3 (42.4–56.2)	43.3 (36.5–50.2)	36.8 (29.7–43.9)
Treatment 3	37 (23.6%)	56.6 (49.5–63.7)	50.2 (43.5–56.9)	44.0 (37.8–50.3)	39.6 (32.6–46.6)
		**Day 1 1st Entry**	**Day 1 2nd Entry**	**Day 1 3rd Entry**	**Day 1 4th Entry**
Group	Count (Percent)	Median (95% CI)	Median (95% CI)	Median (95% CI)	Median (95% CI)
Placebo	35 (22.3%)	55.2 (46.8–63.6)	58.4 (49.4–67.3)	52.9 (43.2–62.6)	50.0 (39.1–60.9)
Treatment 1	43 (27.4%)	53.5 (42.8–64.2)	43.2 (30.4–55.9)	38.8 (24.0–53.6)	27.9 (16.1–39.7)
Treatment 2	42 (26.8%)	57.6 (42.9–71.7)	50.0 (39.1–60.9)	49.9 (37.0–62.7)	42.4 (28.0–56.9)
Treatment 3	37 (23.6%)	50.4 (39.3–61.5)	47.5 (41.1–53.8)	41.7 (34.5–48.9)	37.4 (25.0–49.8)

**Table 3:** Descriptive statistics for STPIS measurements on Day 2. Survey data for each participant was collected upon waking and every four hours thereafter during waking hours.

		**Day 2 1st Entry**	**Day 2 2nd Entry**	**Day 2 3rd Entry**	**Day 2 4th Entry**
Group	Count (Percent)	Mean (95% Cl)	Mean (95% Cl)	Mean (95% Cl)	Mean (95% Cl)
Placebo	35 (22.29%)	51.4 (43.2–59.5)	48.7 (40.8–56.6)	45.8 (37.5–54.1)	47.5 (38.4–56.7)
Treatment 1	43 (27.89%)	34.1 (26.9–41.8)	28.0 (21.7–34.4)	22.7 (16.9–25.6)	14.4 (9.7–19.1)
Treatment 2	42 (26.75%)	34.7 (28.1–41.3)	26.2 (19.8–22.6)	22.6 (16.3–28.9)	18.0 (11.8–24.2)
Treatment 3	37 (23.57%)	35.5 (28.4–42.5)	27.0 (20.9–33.1)	20.4 (15.6–25.1)	12.4 (7.9–16.9)
		**Day 2 1st Entry**	**Day 2 2nd Entry**	**Day 2 3rd Entry**	**Day 2 4th Entry**
Group	Count (Percent)	Median (95% CI)	Median (95% CI)	Median (95% CI)	Median (95% CI)
Placebo	35 (22.29%)	50.0 (42.8–57.2)	50.0 (44.3–55.7)	49.5 (39.8–59.2)	50.5 (42.0–59.0)
Treatment 1	43 (27.89%)	31.7 (19.9–43.6)	23.0 (12.8–33.1)	19.1 (13.8–24.4)	7.9 (2.1–13.6)
Treatment 2	42 (26.75%)	36.2 (25.7–47.0)	24.4 (17.0–31.7)	18.3 (9.9–26.7)	10.8 (3.4–18.3)
Treatment 3	37 (23.57%)	40.0 (30.1–49.9)	25.3 (15.1–35.5)	17.4 (12.0–22.9)	10.1 (5.4–14.9)

Changes in STPIS assessments between Day 1 and Day 2 are shown in Tables 4 and 5.

**Table 4:** Day 1 changes in assessment score. Changes in assessment scores taken on Day 1 were compared to the first assessment (before treatment initiation); survey data for each participant was collected upon waking and every four hours thereafter during waking hours. Overall differences in mean changes in STPIS among all four groups were measured.

	Day 1 Changes in Assessment Score					
	Δ 2nd vs.1st		Δ 3rd vs. 1st		Δ 4th vs. 1st	
Group	Mean (95% Cl)	P-Value	Mean (95% Cl)	P-Value	Mean (95% Cl)	P-Value
Placebo	-0.8 (-3.4–1.7)		-4.8 (-7.7 - -1.9)		-10.7 (-16.2 - -5.3)	
Treatment 1	-9.9 (-13.1 - -6.6)	0.005	-17.0 (-21.0- -13.1)	0.002	-23.0 (-28.1 - -17.8)	0.028
Treatment 2	-7.3(-11.4–3.2)		-13.3 (-17.7 - -8.9)		-19.8 (-25.5 - -14.1)	
Treatment 3	-6.4 (-10.2 - -2.6)		-12.6 (-18.3 - -6.9)		-17.0 (-24.1 - -9.9)	
Group	**Δ 2nd vs.1st** Median (95% CI)	**Δ 3rd vs. 1st** Median (95% CI)	**Δ 4th vs. 1st** Median (95% CI)
Placebo	-0.8 (-3.0–1.7)		-4.1 (-7.3 - -0.9)		-8.6 (-14.6 - -2.5)	
Treatment 1	-5.6 (-11.0 - -0.2)		-15.2 (-20.5 - -9.9)		-18.7 (-24.8 - -12.6)	
Treatment 2	-2.4(-5.7–0.8)		-9.6 (-16.1 - -3.1)		-16.3 (-23.9 - -8.8)	
Treatment 3	-5.2(-9.4 - -0.9)		-10.9 (-15.9 - -5.8)		-15.9 (-24.6 - -7.3)	

**Table 5:** Day 2 Changes in assessment score. Changes in assessment scores taken on Day 2 were compared to the first assessment (before treatment initiation); survey data for each participant was collected upon waking and every four hours during waking hours. Overall differences among all four groups in STPIS mean changes from Day 1 to Day 2 were measured.

	Day 2 Changes in Assessment Score							
	Δ 5th vs.1st		Δ 6th vs.1st		Δ 7th vs.1st		Δ 8th vs.1st	
Group	Mean (95% Cl)	P-Value	Mean (95% Cl)	P-Value	Mean (95% Cl)	P-Value	Mean (95% Cl)	P-Value
Placebo	-4.0 (-8.8–0.8)		-6.7 (-11.7 - -1.6)		-9.6 (-15.4 - -3.8)		-7.8 (-14.2 - -1.5)	
Treatment 1	-22.7 (-27.9 - -17.5)	< .0001	-28.8 (-33.7 - -23.8)	< .0001	-34.1 (-39.6 - -28.6)	< .0001	-42.4 (-48.3 - -36.5)	< .0001
Treatment 2	-21.9 (-27.8- -16.0)		-30.4 (-36.6 - -24.3)		-34.0 (-40.4 - -27.7)		-38.6 (-45.6 - -31.6)	
Treatment 3	-21.1 (-27.3- -15.0)		-29.6 (-36.4 - -22.8)		-36.2 (-43.4 - -29.1)		-44.2 (-52.1 - -36.3)	
Group	**Δ 5th vs.1st** Median (95% CI)		**Δ 6th vs.1st** Median (95% CI)		**Δ 7th vs.1st** Median (95% CI)		**Δ 8th vs.1st** Median (95% CI)	
Placebo	-3.3 (-8.1–1.5)		-5.2 (-9.5 - -0.9)		-7.9 (-13.5 - -2.3)		-6.3 (-14.0–1.4)	
Treatment 1	-20.0 (-24.9 - -15.1)		-25.4 (-31.3 - -19.5)		-29.7 (-37.2 - -22.2)		-39.7 (-47.1 - -32.4)	
Treatment 2	-22.0 (-29.7 - -14.3)		-29.2 (-33.0 - -25.4)		-33.4 (-38.6 - -28.2)		-39.7 (-45.6 - -33.7)	
Treatment 3	-20.0 (-27.2 - -12.8)		-22.73 (-31.0 - -14.5)		-33.4 (-45.2 - -21.6)		-40.5 (-51.8 - -29.3)	

**Fig 2:**
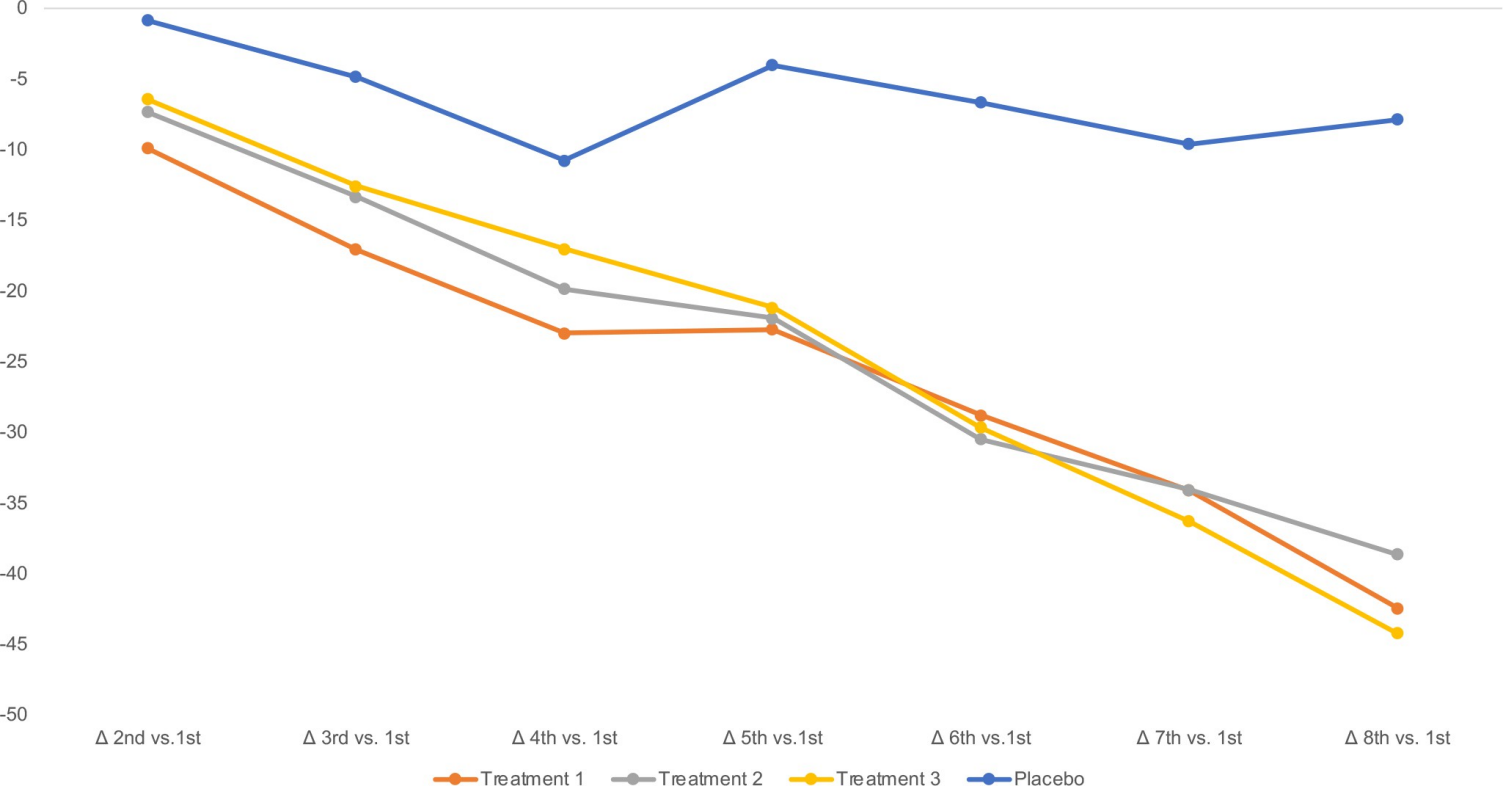
Changes in the STPIS means by group. Changes in the mean STPIS score at the first assessment on Day 1 to the last assessment on Day 2 (the primary endpoint) are shown for each treatment group. Numerical values of those graphed can be seen in Table 5.

The changes on Day 2 were greater than changes on Day 1 and the significance of the differences between the groups was greater on Day 2 than on Day 1. Change in STPIS score from the fourth measure of the second day compared to the first measure on the first day was the primary endpoint of the trial. STPIS Day 2, 4^th^ entry (36 hours after treatment initiation), was statistically significant from baseline (1^st^ entry) for each of the treatments (p< 0.0001) as well as placebo (Table 3). The mean changes were placebo (-7.84 [95% CI -14.20 to -1.47]; p<0.0001) (-14%), Treatment 1 (-42.41 [95% CI -48.30 to -36.52]; p<0.0001) (-68%), Treatment 2 (-38.60 [95% CI -46.64 to -31.56]; p<0.0001) (-75%), and Treatment 3 (-44.19 [95% CI -52.11to -36.27]; p<0.0001) (Table 5 and Fig 2).

Additional post hoc analyses (pairwise comparisons) shown in Table 6 found statistically significant differences between placebo and each of the treatments (p< 0.0001) but not between the treatments themselves.

**Table 6:** Post-hoc analysis showing pairwise comparisons of changes in assessment scores between groups.

	Comparison Between Groups												
		Δ 5th vs.1st			Δ 6th vs.1st			Δ 7th vs.1st				Δ 8th vs.1st	
Contrast	DF	Mean Square	F Value	Pr> F	Mean Square	F Value	Pr> F	Mean Square	F Value	Pr> F	Mean Square	F Value	Pr> F
Placebo vs Treatment 1	1	6,739.38	22.7	< .0001	9,419.92	29.3	< .0001	11,559.14	31.2	< .0001	23,058.42	51.7	< .0001
Placebo vs Treatment 2	1	6,113.48	20.5	< .0001	10,807.70	33.6	< .0001	11,416.47	30.8	< .0001	18,068.80	40.5	< .0001
Placebo vs Treatment 3	1	5,291.43	17.8	< .0001	9,500.80	29.6	< .0001	12,781.85	34.5	< .0001	23,766.88	53.3	< .0001
Treatment 1 vs Treatment 3	1	46.98	0.16	0.692	15.68	0.05	0.826	94.68	0.26	0.614	63.12	0.14	0.707
Treatment 2 vs Treatment 3	1	10.85	0.04	0.849	12.91	0.04	0.841	95.54	0.26	0.612	613.90	1.38	0.243
Treatment 1 vs Treatment 2	1	13.40	0.1	0.832	61.24	0.2	0.663	0.01	0.0	0.996	307.61	0.7	0.408

### Secondary outcomes

**Fig 3:**
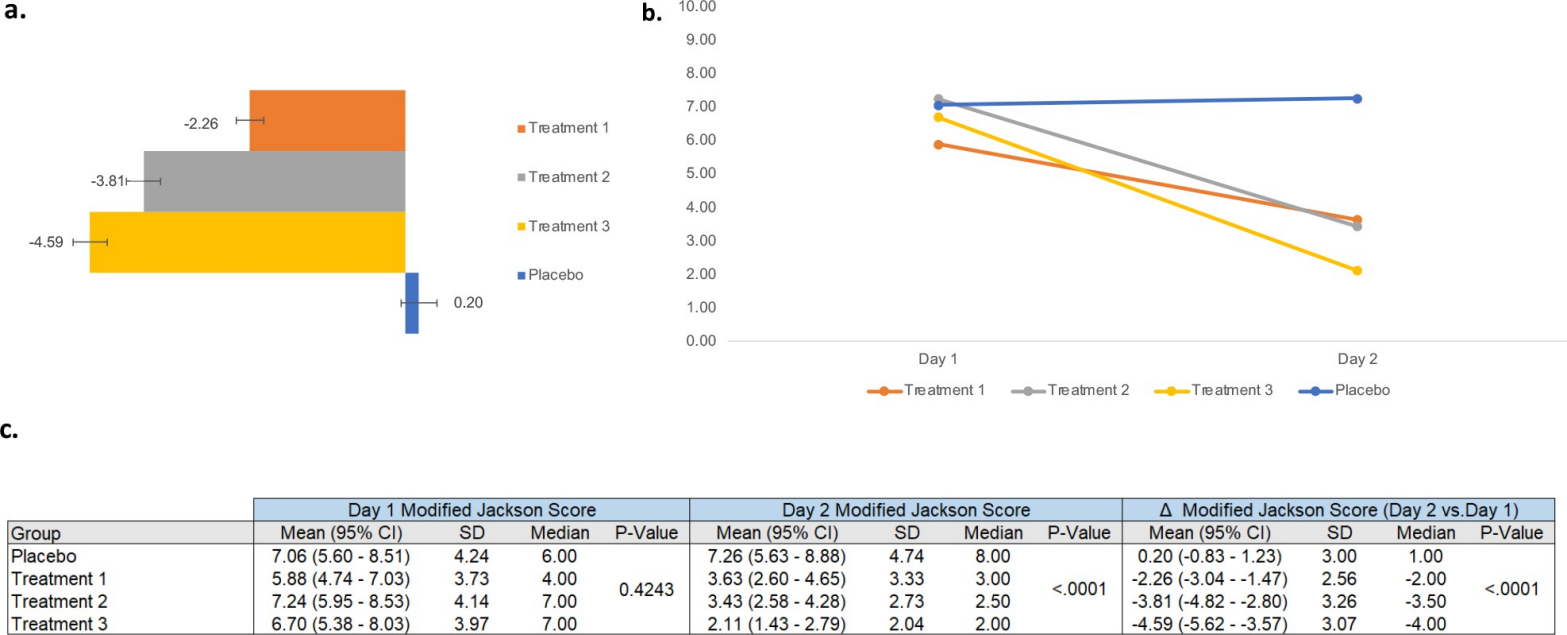
Assessment of Jackson scores. Mean Jackson scores on Day 1 and Day 2 are shown for each treatment group in a bar graph along with the numerical value for the mean and an error bar depicting the standard error of the mean (a). The same values are also shown in a line graph (b). Statistical analyses are also shown (c).

Effects on the eight sets of Jackson Score measures were analyzed using repeated measures analysis to measure within subject changes on MJS. MJS includes a symptom severity questionnaire for eight symptoms: sneezing, nasal discharge, nasal congestion, sore/scratchy throat, cough, headache, malaise and fever/chills. Each is from 0 to 3 (0 = absent, 1 = mild, 2 = moderate, 3 = severe) [52]. Significant differences on MJS were found between Treatments on Day 2 (p < 0.0001) (Fig 3A–3C). Treatment 3 exhibited the largest mean change, indicating the most significant improvement in symptom severity compared to the other groups (Fig 3A–3C) (-4.59 [95% CI -5.62 to -3.57]; p<0.0001). Post hoc pairwise comparisons revealed significant differences between Treatment 1 and Treatment 3, as well as between Treatment 1 and Treatment 2, each treatment and placebo (p<0.0001) (Fig 3C). Individually, each of the eight symptom scores showed some significant differences between groups on Day 2.

**Fig 4:**
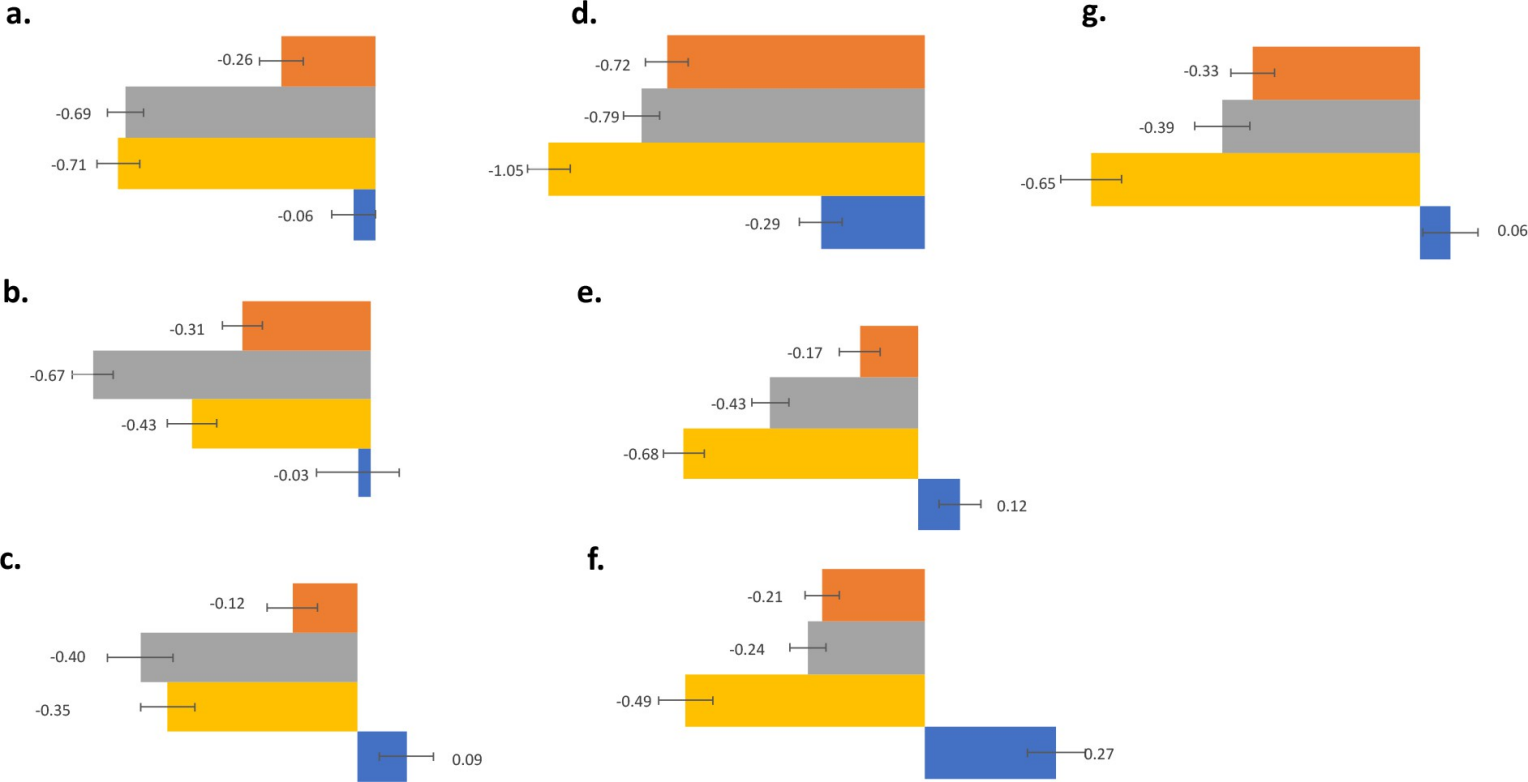
Assessment of individual symptoms. Changes in mean scores for individual symptoms from Day 2 to Day 1 for each treatment group are shown in a bar graph along with numerical values and an error bar depicting standard error of the mean: Nasal congestion (a), nasal discharge (b), sneezing (c), sore throat (d), cough (e), headache (f), and malaise (g).

For nasal congestion, Treatments 2 and 3 were significantly better than placebo and Treatment 1 but not different from each other (Fig 4A). From Day 1 to Day 2 the mean score for Treatment 1 declined by 0.26 points (27.6%) compared to 0.06 points (5.5%) for placebo. For Treatments 2 and 3 nasal congestion decreased by 0.69 points (57%) and 0.71 points (66.7%) from Day 1–2.

For nasal discharge, Treatment 2 produced the strongest effect (Fig 4B). The effect of Treatment 2 was statistically better than placebo and also Treatment 1. The effects of Treatment 3 compared to placebo were also significant albeit showed less decrease than Treatment 2. The differences between Treatments 2 and 3 were not statistically significant. Treatments 1–3 decreased nasal discharge scores by 0.31 (42.1%), 0.67 (59.2%), and 0.43 (58.1%) respectively.

For sneezing, Treatments 2 and 3 produced the strongest effects (Fig 4C). The effect of Treatment 2 and 3 were statistically better than placebo. The differences between Treatments 2 and 3 were not statistically significant. Treatments 1–3 decreased sneezing scores by 0.12 (34%), 0.4 (47.7%), and 0.35 (56.5%) respectively. The effects of Treatment 1 were not significantly different from that of placebo for any of the nasal symptoms. Both Treatments 2 and 3 used throat sprays containing wintergreen oil which may have contributed to decreasing all nasal symptoms, however aspirin did not seem to provide any additional benefit on this measure.

Participants in the placebo group reported a decrease of 17.5% in sore throat scores between Day 2 and Day 1. Nevertheless, all treatments showed statistically significant decreases compared to placebo with Treatment 3 showing the largest decrease (Fig 4D). The effect of Treatment 3 was statistically different from Treatment 1 but not Treatment 2. Treatments 1–3 decreased sore throat scores by 0.72 (45%), 0.79 (53.5%), and 1.05 (66%) respectively. The results of the sore throat Jackson Score measure showed similar trends as seen with STPIS, however differences among the groups were not statistically different for STPIS. This may be explained by the STPIS measuring changes from the initiation of treatment (morning of Day 1) whereas the Jackson Score measured change from Day 1 (end of day) to Day 2 (end of day), or that the STPIS included more frequent measures (12 times a day versus 1 time a day).

Treatment 3 had the strongest effect on cough (Fig 4E). Treatment 3 was statistically different from placebo and Treatment 1, but not from Treatment 2. Treatment 2 also showed statistically significant improvement compared to placebo. Treatment 1 was not statistically significant from placebo (Fig 4D). Treatments 1–3 decreased cough scores by 0.17 (23.4%), 0.43 (46.2%), and 0.68 (71.6%) respectively. Both Treatments 2 and 3 used throat sprays containing wintergreen oil which may have contributed to decreasing cough symptoms. Aspirin may provide some additional benefit on this measure.

All treatments showed a statistically significant improvement in headache and malaise scores compared to placebo (Fig 4F and 4G). Treatment 3 had the strongest effect and was significantly better than Treatment 1 but not Treatment 2. (Fig 4F and 4G), Treatments 1–3 decreased headache by 0.21 (44.7%) and 0.33 (48.4%), 0.24 (48%) and malaise by 0.39 (50%), 0.49 (77.4%) and 0.65 (77.4%) respectively.

## Discussion

In this double-blind placebo-controlled randomized clinical trial, we tested three novel treatments against placebo for alleviating common cold symptoms. Outcomes were measured using two clinically validated scales: the STPIS measured sore throat pain and the Jackson score measured eight different cold symptoms including congestion, nasal discharge, sneezing, and others. All three treatments showed statistically significant differences compared to placebo on both scales. STPIS decreased 68–75% by 36 hours depending on treatment. Nasal discharge, congestion, sneezing, cough, sore throat, and malaise decreased 38–68% depending on treatment. According to our knowledge of the literature, these are the greatest decreases to date in overall illness severity using clinically validated measures and comparable study design.

We treated respiratory symptoms by both strengthening the respiratory epithelia and reducing inflammation. Inflammation is a protective response meant to recruit immune modulators, yet excessive inflammation can damage respiratory epithelia increasing the likelihood of secondary infection, and the duration of respiratory symptoms [54]. Thus, we reasoned, reducing inflammation and strengthening the respiratory epithelia are both necessary to limit disease severity. Wintergreen oil and aspirin contain salicylates (methylsalicylate and acetylsalicylate respectively), which decrease inflammation via inhibition of COX enzymes. COX enzymes convert arachidonic acid to prostaglandins; immune modulators responsible for common cold symptoms [32–36]. According to a previously published study using human respiratory organoids, acetylsalicylic acid was able to prevent increases in prostaglandin levels mediated by bradykinin and COX enzymes [39]. This inhibition as well as membrane integrity were enhanced when acetyl salicylic acid was mixed with a formula containing lysozyme, lactoferrin, and aloe [39].

In this study we treated 157 participants having common cold symptoms with a MIC throat spray containing lysozyme, lactoferrin, and aloe to strengthen the respiratory lining as well as menthol, a commonly used analgesic and either wintergreen oil or aspirin to treat inflammation (Treatments 1–3). Compared to placebo spray, all treatment groups showed significant improvement in sore throat and other common cold symptoms such as cough, congestion, and sneezing. The study successfully met both its primary and secondary endpoints. STPIS began to improve upon the first treatment (Table 4 and Fig 2). By 36 hours, STPIS decreased 68–79% depending on treatment (Table 4 and Fig 2). The MJS which is comprised of eight symptoms, decreased 38% for Treatment 1, 52.6% for Treatment 2, and 68% for Treatment 3 between the first and second day after treatment initiation (Fig 3). On between group comparison for MJS from Day 1 to Day 2, Treatments 2 and 3 performed significantly better than Treatment 1.

Aspirin on its own has been studied previously for treating common cold symptoms, but its effects were not as strong as those for our throat spray paired with aspirin (Treatment 3) or the spray alone (Treatment 2). A previous study of 800 mg aspirin paired with vitamin C showed a decrease in Wisconsin Upper Respiratory Symptom Survey Domain 2, a validated scale of common cold symptoms of 29% compared to placebo 2 hours after treatment initiation, a decrease of 30.2% at the end of the first day of treatment, and a decrease of only 12% by the second day [31]. Effects on Days 3 and 4 were not statistically different from placebo [31]. In another study where patients were given 800 mg of aspirin, and assessed for 6 hours after treatment, sore throat pain intensity differences decreased 58% 2 hours after treatment [55]. In the same study, 6 hours after treatment, headache was reduced 38% compared to 16% for placebo, muscle aches and pains were reduced 38% following treatment and 25% following placebo. Differences in sinus pain and fever were not different from placebo at 6 hours post treatment [55]. While this study was not designed to directly compare the test treatments to individual COX-inhibitors, such as aspirin, when compared to previous studies, our results suggest that the combination of a COX-inhibitor with MIC may be more effective than a COX-inhibitor alone.

Even Treatment 1, the least effective treatment containing 6 mg of aspirin, decreased common cold symptoms more than previously observed with 800 mg aspirin. Unexpectedly, an equivalent amount (6 mg) of wintergreen oil (Treatment 2) in lieu of aspirin led to a greater improvement in nasal symptoms, but not in pain-associated symptoms (Fig 4). The addition of an aspirin pill to the wintergreen oil (Treatment 3) did not further improve nasal symptoms, but did improve pain-associated symptoms (Fig 4). This demonstrates that wintergreen oil offers an additional benefit for nasal symptoms, that commonly arise during upper respiratory tract infections. The wintergreen oil in Treatment 2 could be acting by several mechanisms and may explain the stronger efficacy of Treatment 2. In addition to methyl salicylate, wintergreen contains numerous essential oils, a mix of aldehydes, esters, ketones, peroxides, and phenols that can be inhaled and carried throughout the respiratory tract. These have been shown to have antimicrobial and anti-inflammatory effects [56]. They may act on TRP ion channels in the airways which play a role in respiratory symptoms [56]. Menthol also acts on TRP ion channels and is composed of a unique mix of terpenoids to wintergreen oil. It may have additive or synergistic effects when combined with wintergreen oil [56]. Formulations in this study were made with natural menthol and wintergreen oil. Chemical compositions may be influenced by environmental factors and isolation processes, which should be standardized in future studies. The absence of treatment groups receiving aspirin or other components alone limits our ability to quantitate the contribution of each component in the formula individually. Here we compared effects of our treatments to previously published effects of aspirin. Future studies should include treatments with aspirin alone to more directly compare the effects of aspirin with or without MIC, and should also control for the effects of wintergreen oil and menthol.

In summary, this randomized, double-blind, placebo-controlled trial tested the effects of three different treatments on common cold symptoms. All three treatments combine an epithelium-protecting MIC with a COX inhibitor. All three tested treatments demonstrated strong efficacy for reducing multiple cold symptoms. The extent of symptom reduction exceeded the previously published effects of COX inhibitors or other anti-inflammatory treatments.

## Supporting information

S1 File Checklist of information to include when reporting a randomized control trial. (DOCX)

S2 File Treatment groups and survey forms. (PDF)

S3 File Data management report. (PDF)
