# Supplementary material for: Supporting respiratory epithelia and lowering inflammation to effectively treat common cold symptoms: A randomized controlled trial
Source: PLoS One. 2024 Nov 27;19(11):e0301959. doi: 10.1371/journal.pone.0301959 (PMC11602100; doi:10.1371/journal.pone.0301959)
Supplement: S2 File — (PDF) [file pone.0301959.s002.pdf]

ECONOMETRICA, INC.

Biovanta Clinical Trial Analysis Report

Biovanta Sore Throat Study

Project No.: 2944-000

Submitted To:

Applied Biological Laboratories
New York Genome Center
101 Avenue of Americas, 3rd Floor
New York, NY 10013
[redacted]

June 16, 2023

25TH ANNIVERSARY 1998–2023

Econometrica, Inc.
7475 Wisconsin Avenue, Suite 1000 | Bethesda, MD 20814 | Phone: (301) 657-9883 | Fax: (301) 657-3140
www.EconometricaInc.com | DUNS Number: 196693170 | TIN: 52-2108043 | Cage Code: 1PZY5 | UEI: EHJKHN7FE8D7
GSA Multiple Award Schedules No. GS-00F-101CA and GS-35F-355CA

ECONOMETRICA, INC.

25TH ANNIVERSARY 1998–2023

June 16, 2023

Applied Biological Laboratories
New York Genome Center
101 Avenue of Americas, 3rd Floor
New York, NY 10013
[redacted]

Reference: "Biovanta Sore Throat Study" (Project No. 2944-000)

Econometrica is pleased to submit this Biovanta Clinical Trial Analysis Report, produced under the above-referenced contract.

If you wish to discuss any aspect of this submission, please feel free to contact me at [redacted] or [redacted]

Sincerely,

Econometrica, Inc.

[redacted]
Project Director
[redacted]

Cc: Contract File

Table of Contents

LIST OF TABLES .... **IV**

LIST OF FIGURES .... **IV**

LIST OF ACRONYMS .... **VI**

1. BACKGROUND **1**

2. BIOVANTA SURVEY DATA **3**

2.1. HANDLING PATIENT FORMS 3

2.2. DATA PROCESSING 3

3. STATISTICAL ANALYSIS **6**

3.1. ANALYSIS OF STPIS AND JACKSON SCORE RESPONSES 6

3.2. ANALYSIS OF "DO YOU STILL HAVE A COLD?" RESPONSES 6

4. FINDINGS **7**

4.1. FINDINGS HIGHLIGHTS 7

4.2. DETAILED RESULTS 8

Analytic Excellence, Innovative Solutions, Quality Service

List of Tables

TABLE 1. RE-CODED “DO YOU STILL HAVE A COLD?” VALUES 5

List of Figures

FIGURE 1: SAMPLE STPIS RESPONSE... 3
FIGURE 2: SAMPLE JACKSON SCORE ASSESSMENT TABLE 4
FIGURE 3: SAMPLE "DO YOU STILL HAVE A COLD?" RESPONSE 5
FIGURE 4: STPIS DESCRIPTIVE STATISTICS – DAY 1... 8
FIGURE 5: STPIS DESCRIPTIVE STATISTICS – DAY 2... 9
FIGURE 6: REPEATED MEASURES MULTIVARIATE ANOVA TEST... 9
FIGURE 7: CHANGES IN STPIS ASSESSMENTS IN DAY 1 10
FIGURE 8: CHANGES IN STPIS ASSESSMENTS IN DAY 2 10
FIGURE 9: PAIRWISE COMPARISON OF STPIS CHANGES IN DAY 1 10
FIGURE 10: PAIRWISE COMPARISON OF STPIS CHANGES IN DAY 2 11
FIGURE 11: CHANGES IN STPIS MEANS BY GROUP 11
FIGURE 12: MULTIVARIATE ANOVA TEST FOR THE MODIFIED JACKSON SCORE 12
FIGURE 13: SIGNIFICANCE TEST AND DAY 1 VS. DAY 2 CHANGES IN THE MODIFIED JACKSON SCORES 13
FIGURE 14: MEAN OF CHANGES IN THE MODIFIED JACKSON SCORES ACROSS THE DIFFERENT GROUPS... 13
FIGURE 15: PAIRWISE COMPARISON ANALYSIS OF CHANGES IN THE MODIFIED JACKSON SCORES.14
FIGURE 16: MULTIVARIATE ANOVA TEST FOR THE NASAL CONGESTION SYMPTOM 14
FIGURE 17: SIGNIFICANCE TEST AND DAY 1 VS. DAY 2 CHANGES IN THE NASAL CONGESTION SYMPTOM.. 15
FIGURE 18: MEAN OF CHANGES IN THE NASAL CONGESTION SYMPTOM ACROSS THE DIFFERENT GROUPS... 15
FIGURE 19: PAIRWISE COMPARISON ANALYSIS OF CHANGES IN THE NASAL CONGESTION SYMPTOM.. 16
FIGURE 20: MULTIVARIATE ANOVA TEST FOR THE NASAL DISCHARGE SYMPTOM....................... 16
FIGURE 21: SIGNIFICANCE TEST AND DAY 1 VS. DAY 2 CHANGES IN THE NASAL DISCHARGE SYMPTOM.. 17
FIGURE 22: MEAN OF CHANGES IN THE NASAL DISCHARGE SYMPTOM ACROSS THE DIFFERENT GROUPS... 17
FIGURE 23: PAIRWISE COMPARISON ANALYSIS OF CHANGES IN THE NASAL DISCHARGE SYMPTOM.. 18

Figure 24: Multivariate ANOVA Test for the Sneezing Symptom 18
Figure 25: Significance Test and Day 1 vs. Day 2 Changes in the Sneezing Symptom 18
Figure 26: Mean of Changes in the Sneezing Symptom Across the Different Groups..... 19
Figure 27: Pairwise Comparison Analysis of Changes in the Sneezing Symptom 19
Figure 28: Multivariate ANOVA Test for the Sore Throat Symptom 20
Figure 29: Significance Test and Day 1 vs. Day 2 Changes in the Sore Throat Symptom.......... 20
Figure 30: Mean of Changes in the Sore Throat Symptom Across the Different Groups.......... 20
Figure 31: Pairwise Comparison Analysis of Changes in the Sore Throat Symptom....... 21
Figure 32: Multivariate ANOVA Test for the Cough Symptom 21
Figure 33: Significance Test and Day 1 vs. Day 2 Changes in the Cough Symptom 21
Figure 34: Mean of Changes in the Cough Symptom Across the Different Groups......... 22
Figure 35: Pairwise Comparison Analysis of Changes in the Cough Symptom 22
Figure 36: Multivariate ANOVA Test for the Headache Symptom 23
Figure 37: Significance Test and Day 1 vs. Day 2 Changes in the Headache Symptom 23
Figure 38: Mean of Changes in the Headache Symptom Across the Different Groups... 23
Figure 39: Pairwise Comparison Analysis of Changes in the Headache Symptom 24
Figure 40: Multivariate ANOVA Test for the Malaise Symptom 24
Figure 41: Significance Test and Day 1 vs. Day 2 Changes in the Malaise Symptom 24
Figure 42: Mean of Changes in the Malaise Symptom Across the Different Groups...... 25
Figure 43: Pairwise Comparison Analysis of Changes in the Malaise Symptom 25
Figure 44: Multivariate ANOVA Test for the Fever/Chills Symptom.............................. 26
Figure 45: Significance Test and Day 1 vs. Day 2 Changes in the Fever/Chills Symptom.......... 26
Figure 46: Mean of Changes in the Fever/Chills Symptom Across the Different Groups.......... 26
Figure 47: Pairwise Comparison Analysis of Changes in the Fever/Chills Symptom...... 27
Figure 48: Chi-square Frequency of "Do you still have a cold?" – Day 1......................... 27
Figure 49: Chi-square Frequency of "Do you still have a cold?" – Day 2......................... 27
Figure 50: Fisher's Test Frequency Table 28

List of Acronyms

Acronym	Definition
ANOVA	Analysis of Variance
CI	Confidence Interval
PDF	Portable Document Format
PI	Principal Investigator
RDBPC	Randomized Double-Blind Placebo Control Study
STPIS	Score Throat Pain Intensity Scale

1. Background

To evaluate the effectiveness of its new cold and sore throat medicine Biovanta, Applied Biological Laboratories (Applied Bio) conducted a randomized double-blind placebo control (RDBPC) study on 179 participants. The study participants were recruited from multiple locations (Atlanta, Baltimore, Houston, New York, Washington, DC, and Orange County, CA). Individuals with cold symptoms were directed to administer Biovanta over the course of two days while completing patient forms assessing their sore throat pain and cold symptoms throughout the course of the treatment.

The following are the roles of various entities and individuals in the clinical trial and their blind status:

Responsibility	Organization / Individual	Blind Status
Internal Review Board:	ADVARRA	
Clinical Trials CRO:	[redacted]	Blinded
Principal Investigator:	[redacted], DO	Blind
Medical Services:	[redacted] Medical Associates	
	[redacted] M.D.	Blind
	[redacted]M.D.	Blind
	[redacted] M.D.	Blind
	[redacted]M.D.	Blind
	[redacted]M.D.	Blind
	[redacted]D.O. (PI)	Blind
Manufacturer	[redacted]abs	Unblind
	[redacted]nt)	Signed Declaration Form
	[redacted]	Signed Declaration Form
	[redacted]	Signed Declaration Form
	[redacted]	Signed Declaration Form
Clinical Trials Coordinator	[redacted]	Blind
	[redacted]	Blind
Clinical Trials Administrators	[redacted]RN	Blind
	[redacted]RN	Blind
	[redacted]MD	Blind
	[redacted]	Blind
	[redacted]	Blind
	[redacted]RN	Blind
	[redacted]e LPN	Blind
	[redacted]CAN	Blind
Participant Recruitment	Splash Clinical	Blind
	Seasons	Blind
Adverse Event Specialist	[redacted]MD	Unblind with Signed Declaration Form
Data Analytics	Econometrica	
	[redacted]	Blind but blind access to crosswalk
	[redacted]	Blind but blind access to crosswalk
	[redacted]	Blind but blind access to crosswalk
	[redacted]	Blind but blind access to crosswalk

The study comprised the following clinical trial groups:

- Group A: Placebo
- Group B: Treatment 3
- Group C: Treatment 1
- Group D: Treatment 2

Applied Bio contracted Econometrica, Inc. (Econometrica) to perform the analysis of the Biovanta sore throat survey data. This report details Econometrica's analysis of the clinical trial data and our findings. This report is organized as follows:

- The **Biovanta Survey Data** section discusses the survey data Econometrica received from Applied Bio and the steps taken to import, re-code, clean, and validate the data.
- The **Statistical Analysis** section lists the types of analyses Econometrica performed on the data.
- The **Findings** section contains the results of the study.

2. Biovanta Survey Data

2.1. Handling Patient Forms

Montgomery Medical Associates, led by Aushiravan Dadgar, D.O., provided Econometrica with 159 paper patient forms filled out by study participants. Each form contained participants' responses to questions concerning sore throat pain intensity scale (STPIS), Jackson score assessment, and cold symptoms, for both days of the study.

Econometrica staff scanned the paper forms into an electronic PDF format. (The scanning was performed in the presence of Dr. Dadgar, who then collected the original forms.) Next, the electronic forms were transferred to Econometrica's secure HIPAA-compliant analytical server, where data processing and analysis took place.

Econometrica has not shared the patient forms or individual participant responses with anyone outside of the Econometrica team involved in this analysis.

2.2. Data Processing

This section describes the steps Econometrica took to clean and prepare the patient data for statistical analysis.

2.2.1. Data Entry and Re-Coding

Econometrica performed the following steps to import, measure, and clean the data:

- **Processing STPIS values**:
  - Study participants provided their STPIS responses by marking the STPIS line to indicate the intensity of their sore throat pain, ranging from "no pain" to "severe pain," as shown in Figure 1.

    Figure 1: Sample STPIS Response

    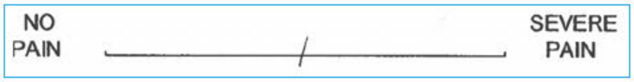

  - Because the STPIS lines did not have a numeric scale, Econometrica converted participants' markings into numeric values. The marking-to-number conversion was performed as follows:
    - Econometrica used Adobe's Measuring Tool to measure the length of the entire STPIS line. This value became the denominator for the STPIS measure.
    - We then measured the distance from the beginning of the STPIS line to the mark inscribed by the participant. This value became the numerator for the STPIS measure.
    - The division of the numerator by the denominator gave us the STPIS value, which we adjusted to a 100-point scale (0 being no pain and 100 being the most severe pain). For instance, if the length of the entire STPIS line in the patient form was 3.65 inches (the denominator) and the distance to the STPIS mark made by the

respondent was 2.70 inches, the STPIS value was determined to be 2.70 / 3.65 = .7397, or 73.97 points when adjusted to a 100-point scale.

- A few participants wrote numbers above the STPIS lines, in addition to or instead of marking the lines. In these cases, we used the numbers provided by the participants (and adjusted to a 100-point scale, if necessary) as the STPIS values. For example, an "8" written by a respondent became an STPIS value of 80.

- **Processing Jackson Score Assessment values**:
  - The Jackson Score Assessment table asked respondents to check "Absent," "Mild," "Moderate," or "Severe" indicators for eight cold-related symptoms, as shown in Figure 2.

Figure 2: Sample Jackson Score Assessment Table

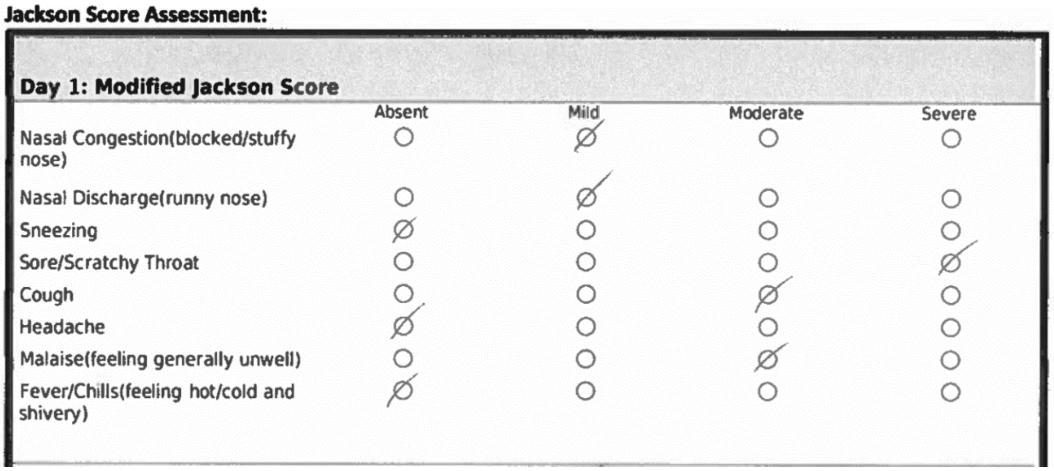

Jackson Score Assessment:

Day 1: Modified Jackson Score

	Absent	Mild	Moderate	Severe
Nasal Congestion(blocked/stuffy nose)	○	⊘	○	○
Nasal Discharge(runny nose)	○	⊘	○	○
Sneezing	⊘	○	○	○
Sore/Scratchy Throat	○	○	○	⊘
Cough	○	○	⊘	○
Headache	⊘	○	○	○
Malaise(feeling generally unwell)	○	○	⊘	○
Fever/Chills(feeling hot/cold and shivery)	⊘	○	○	○

  - To perform a quantitative analysis of the Jackson scores, the categorical responses were re-coded to numeric values, based on the following formula:
    - Absent = 0
    - Mild = 1
    - Moderate = 2
    - Severe = 3
  - Next, the modified Jackson score was calculated as the sum of the numeric values from all eight symptom assessments. The total modified Jackson score can range from 0 to 24, with higher scores indicating a higher symptom burden.

- **Processing the "Do you still have a cold?" responses**:
  - The final analytical piece from the patient forms was the response to the question "Do you still have a cold or feel like you're coming down with a cold?"

Figure 3: Sample "Do you still have a cold?" Response

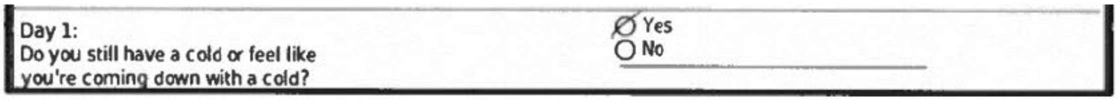
Day 1:
Do you still have a cold or feel like you're coming down with a cold?
Yes
No

- These responses were re-coded to the following categorical values, based on patients' answers across both days of the study:

Table 1. Re-coded "Do you still have a cold?" Values

Day 1 Response	Day 2 Response	Re-Coded Value
"Yes"	"No"	"Better"
"No"	"Yes"	"Worse"
"No"	"No"	"No Cold"
"Yes"	"Yes"	"Same"

2.2.2. Data Cleaning and Integrity Checks

We used the SAS analytical software to perform data cleaning and validation, as well as integrity checks to identify missing, incomplete, incorrect, or suspicious data. We then linked the survey response data to the respondents' respective clinical trial groups, using the participant crosswalk provided by Darius Zandi, M.D., the Adverse Event Specialist.

2.2.3. Excluded Records

Two participants who did not provide information for day 1 or day 2 of the study were excluded from the analysis of the Biovanta sore throat study.

3. Statistical Analysis

This section provides a high-level overview of the statistical analyses Econometrica performed as part of the Biovanta sore throat study. The section **Findings,** below, connects each analysis with its corresponding results.

3.1. Analysis of STPIS and Jackson Score Responses

The following types of analyses were performed on the STPIS and Jackson score responses:

- The mean, 95% confidence interval (CI) of the mean, standard deviation, and median were calculated for each measurement (for both days) to demonstrate the basic characteristics of the data.
- To analyze the changes in scores over time and assess the impact of the interventions, we used the repeated-measures ANOVA. The p-values obtained from the ANOVA tests were used to determine whether the observed differences were statistically significant. In addition, after finding a statistically significant overall impact, we conducted post hoc analysis—pairwise group comparisons—to identify specific differences between each group pair (e.g., A–B, A–C, A–D, B–C, B–D).
- We analyzed patients' changes from their baseline STPIS/Jackson scores to evaluate their progress through the study time points (the baselines were the first STPIS and Jackson score measurements, taken on day 1 of the study). This analysis included the calculation of the mean, 95% CI of the mean, standard deviation, and median, as well as significance tests of the changes for each group. After finding a statistically significant overall impact, we conducted post hoc analysis—pairwise group comparisons—to identify specific differences between each group pair.

3.2. Analysis of "Do you still have a cold?" Responses

Because the re-coded "Do you still have a cold?" values were categorical, we performed the chi-squared test (and Fisher's exact test, to account for fewer respondent counts in the subgroups) to examine the association between the "Do you still have a cold?" variable and the group.

4. Findings

This section details the findings of Econometrica's analysis of the Biovanta sore throat study. The **Findings** section is split into two parts: **Findings Highlights**, which provides an at-a-glance overview of the findings related to the New and Improved Biovanta medicine, and **Detailed Results**, which displays the results of measure- or symptom-specific analyses.

This analysis is based on 157 study participants (the forms from 2 out a total of 159 participants were excluded due to insufficient data). The trial groups were:

- Group A: Placebo
- Group B: Treatment 3
- Group C: Treatment 1
- Group D: Treatment 2

4.1. Findings Highlights

This section highlights the key findings of the Biovanta sore throat study.

- The repeated measures analysis of the overall STPIS and modified Jackson scores (as well as individual Jackson score symptoms) found a statistically significant difference between the intervention groups ($p < 0.05$), indicating that the interventions (drug groups) had varying effects on sore throat pain intensity and other cold symptoms over time.
- The analysis of mean changes in STPIS and modified Jackson scores (as well as individual Jackson score symptoms) also revealed a statistically significant difference among the groups ($p < 0.05$), indicating varying effects of the interventions on reducing throat pain severity and other cold symptoms.
- The post hoc pairwise comparisons of the STPIS changes revealed a statistically significant impact of all Biovanta intervention medicines compared to the placebo group.
- Treatment 3 demonstrated the highest effectiveness in decreasing throat pain severity, as evidenced by the largest mean change in STPIS compared to the other groups.
- Treatment 3 exhibited the largest mean changes in the modified Jackson score, indicating the most significant improvement in symptom severity compared to the other groups.

- The post hoc pairwise comparisons of the modified Jackson scores revealed a statistically significant impact of all Treatments compared to the placebo group. There was also a statistically significant difference between Treatment 3 and Treatment 1.
- Analyses of individual Jackson scores in most instances followed the same patterns as the modified Jackson score, with Treatment 3 demonstrating a strong impact over the other groups in the following categories:
  - Nasal congestion (Treatment 2 and Treatment 3 had the same impact)
  - Sore throat
  - Cough
  - Headache
  - Malaise
  - Fever/chills

4.2. Detailed Results

4.2.1. STPIS Descriptive Statistics

The objective of analyzing STPIS was to evaluate the effects of different drug groups on the intensity of sore throat pain over time. The mean, 95% confidence interval (CI) of the mean, standard deviation, and median for STPIS measures, shown in Figure 4 and Figure 5, are important descriptive statistics that help to summarize and interpret the distribution and central tendency of the data. The mean provides a measure of the central tendency of the data by summarizing the overall magnitude of the sore throat pain intensity scores. The 95% CI of the mean accounts for sampling variability and provides a measure of the precision of the estimated mean. The standard deviation quantifies the variability or dispersion of the STPIS measures around the mean. The median provides an alternative measure of central tendency and is less influenced by extreme values or outliers compared to the mean.

Figure 4: STPIS Descriptive Statistics – Day 1

		Day 1 First Entry			Day 1 Second Entry			Day 1 Third Entry			Day 1 Fourth Entry		
Group	Count (Percent)	Mean (95% CI)	SD	Median	Mean (95% CI)	SD	Median	Mean (95% CI)	SD	Median	Mean (95% CI)	SD	Median
Placebo	35 (22.29%)	55.36 (47.60 - 63.12)	22.59	55.19	54.51 (46.29 - 62.74)	23.95	58.36	50.52 (42.85 - 58.18)	22.32	52.91	44.62 (36.25 - 52.99)	24.36	50.00
Treatment 3	37 (23.57%)	56.61 (49.48 - 63.74)	21.38	50.38	50.19 (43.48 - 56.91)	20.14	47.47	44.04 (37.81 - 50.28)	18.70	41.71	39.60 (32.56 - 46.63)	21.11	37.37
Treatment 1	43 (27.39%)	56.80 (49.88 - 63.72)	22.47	53.49	46.93 (39.79 - 54.07)	23.21	43.15	39.75 (33.04 - 46.47)	21.81	38.83	33.83 (27.16 - 40.49)	21.66	27.91
Treatment 2	42 (26.75%)	56.63 (49.67 - 63.58)	22.32	57.60	49.31 (42.39 - 56.22)	22.19	50.00	43.33 (36.49 - 50.16)	21.94	49.87	36.79 (29.67 - 43.92)	22.86	42.19

Figure 5: STPIS Descriptive Statistics – Day 2

		Day 2 First Entry			Day 2 Second Entry			Day 2 Third Entry			Day 2 Fourth Entry		
Group	Count (Percent)	Mean (95% CI)	SD	Median	Mean (95% CI)	SD	Median	Mean (95% CI)	SD	Median	Mean (95% CI)	SD	Median
Placebo	35 (22.29%)	51.36 (43.24 - 59.47)	23.62	50.00	48.70 (40.75 - 56.64)	23.13	50.00	45.76 (37.48 - 54.05)	24.11	49.45	47.52 (38.35 - 56.69)	26.70	50.52
Treatment 3	37 (23.57%)	35.46 (28.39 - 42.53)	21.21	40.00	26.97 (20.85 - 33.09)	18.35	25.32	20.36 (15.60 - 25.12)	14.28	17.44	12.42 (7.92 - 16.92)	13.50	10.11
Treatment 1	43 (27.39%)	34.11 (26.91 - 41.30)	23.38	31.74	28.04 (21.68 - 34.40)	20.66	22.95	22.73 (16.90 - 28.56)	18.93	19.13	14.39 (9.71 - 19.07)	15.21	7.85
Treatment 2	42 (26.75%)	34.73 (28.07 - 41.39)	21.38	36.24	26.17 (19.80 - 32.55)	20.45	24.36	22.58 (16.30 - 28.85)	20.14	18.29	18.02 (11.84 - 24.21)	19.86	11.03

4.2.2. Repeated Measures Analysis of STPIS Responses

The repeated measures analysis, also known as within-subjects analysis or longitudinal analysis, uses data collected from the same participants over multiple time points, providing increased statistical power compared to cross-sectional designs. By measuring outcomes within the same individuals, the analysis reduces the impact of participant-specific factors that may confound the results, such as genetic variations or individual characteristics. Repeated measures analysis is particularly sensitive to detecting treatment effects over time. It captures the individual trajectory of changes, enabling a more precise evaluation of treatment efficacy and the magnitude of the effect.

We conducted a repeated measures analysis to examine within-subject changes in STPIS across multiple time points. This analysis revealed a statistically significant difference among the intervention groups ($p < 0.05$), indicating that the interventions had varying impacts on sore throat pain intensity over time (see Figure 6).

Figure 6: Repeated Measures Multivariate ANOVA Test

MANOVA Test Criteria and F Approximations for the Hypothesis of STPIS * Group				
Statistic	Value	F Value	Num DF	Pr>F
Wilks's Lambda	0.56	4.48	21	<.0001
Pillai's Trace	0.48	4.02	21	<.0001
Hotelling-Lawley Trace	0.71	4.97	21	<.0001
Roy's Greatest Root	0.61	13.04	7	<.0001

4.2.3. Changes from Baselines

Calculating the changes between each STPIS value and its respective baseline provides a more detailed assessment of changes in pain intensity over time. It takes into account individual variability, controls for baseline differences, and facilitates the evaluation of treatment effects on a participant-specific level. This approach enhances the understanding of how pain intensity evolves and responds to interventions in the context of the study.

We analyzed changes in STPIS means to assess the impact of interventions on reducing throat pain severity (see Figure 7 and Figure 8). The analysis revealed a statistically significant difference in the mean changes in STPIS among the groups ($p < 0.05$), indicating varying

effects of the interventions on reducing throat pain severity. Notably, Treatment 3 demonstrated the highest effectiveness in decreasing throat pain severity, as evidenced by the largest mean change in STPIS compared to the other groups.

Figure 7: Changes in STPIS Assessments in Day 1

	Day 1 Changes in Assessment Score											
	Δ 2nd vs.1st				Δ 3rd vs. 1st				Δ 4th vs. 1st			
Group	Mean (95% CI)	SD	Median	P-Value	Mean (95% CI)	SD	Median	P-Value	Mean (95% CI)	SD	Median	P-Value
Placebo	-0.85 (-3.43 - 1.74)	7.52	-0.76		-4.84 (-7.73 - -1.95)	8.42	-4.11		-10.74 (-16.17 - -5.31)	15.81	-8.57	
Treatment 3	-6.42 (-10.22 - -2.62)	11.41	-5.17	0.0046	-12.57 (-18.26 - -6.88)	17.06	-10.89	0.0015	-17.02 (-24.11 - -9.92)	21.27	-15.95	0.0275
Treatment 1	-9.87 (-13.14 - -6.60)	10.62	-5.57		-17.05 (-21.04 - -13.06)	12.96	-15.19		-22.97 (-28.12 - -17.82)	16.74	-18.73	
Treatment 2	-7.32 (-11.41 - -3.22)	13.14	-2.43		-13.30 (-17.71 - -8.89)	14.14	-9.51		-19.84 (-25.54 - -14.13)	18.31	-16.26	

Figure 8: Changes in STPIS Assessments in Day 2

	Day 2 Changes in Assessment Score															
	Δ 5th vs.1st				Δ 6th vs.1st				Δ 7th vs. 1st				Δ 8th vs. 1st			
Group	Mean (95% CI)	SD	Median	P-Value	Mean (95% CI)	SD	Median	P-Value	Mean (95% CI)	SD	Median	P-Value	Mean (95% CI)	SD	Median	P-Value
Placebo	-4.00 (-8.83 - 0.83)	14.06	-3.29		-6.66 (-11.68 - -1.64)	14.62	-5.21		-9.59 (-15.39 - -3.80)	16.87	-7.95		-7.84 (-14.20 - -1.47)	18.54	-6.31	
Treatment 3	-21.15 (-27.27 - -15.04)	18.33	-20.00	<.0001	-29.64 (-36.45 - -22.84)	20.42	-22.73	<.0001	-36.25 (-43.40 - -29.10)	21.45	-33.42	<.0001	-44.19 (-52.11 - -36.27)	23.75	-40.55	<.0001
Treatment 1	-22.69 (-27.90 - -17.48)	16.94	-20.00		-28.76 (-33.70 - -23.81)	16.06	-25.41		-34.07 (-39.58 - -28.56)	17.91	-29.72		-42.41 (-48.30 - -36.52)	19.13	-39.75	
Treatment 2	-21.90 (-27.79 - -16.01)	18.91	-21.97		-30.45 (-36.62 - -24.29)	19.78	-29.24		-34.05 (-40.38 - -27.71)	20.33	-32.91		-38.60 (-45.64 - -31.56)	22.59	-39.64	

Additional post hoc analyses (pairwise comparisons) found statistically significant differences between placebo and Treatment 3 ($p < 0.05$), between placebo and Treatment 1 ($p < 0.05$), and between placebo and Treatment 2. No significant differences were observed between Treatment 3, Treatment 1, and Treatment 2($p > 0.05$) (see Figure 9 and Figure 10).

These findings suggest that the different drug groups exerted distinct effects on sore throat pain intensity over time, with placebo demonstrating a significantly lower effectiveness compared to the Biovanta medicines.

Figure 9: Pairwise Comparison of STPIS Changes in Day 1

	Day 1												
		Δ 2nd vs.1st				Δ 3rd vs.1st				Δ 4th vs.1st			
Contrast	DF	Contrast SS	Mean Square	F Value	Pr > F	Contrast SS	Mean Square	F Value	Pr > F	Contrast SS	Mean Square	F Value	Pr > F
Compare Placebo & Treatment 3	1	558.67	558.67	4.64	0.0328	1,074.35	1,074.35	5.84	0.0169	708.79	708.79	2.16	0.1441
Compare Placebo & Treatment 1	1	1,571.41	1,571.41	13.05	0.0004	2,873.57	2,873.57	15.62	0.0001	2,886.85	2,886.85	8.78	0.0035
Compare Placebo & Treatment 2	1	799.77	799.77	6.64	0.0109	1,365.79	1,365.79	7.42	0.0072	1,579.56	1,579.56	4.8	0.0299
Compare Treatment 3 & Treatment 1	1	236.88	236.88	1.97	0.1628	398.26	398.26	2.16	0.1433	705.08	705.08	2.14	0.1451
Compare Treatment 3 & Treatment 2	1	15.91	15.91	0.13	0.7168	10.47	10.47	0.06	0.8118	156.27	156.27	0.48	0.4916
Compare Treatment 1 & Treatment 2	1	138.38	138.38	1.15	0.2854	298.06	298.06	1.62	0.205	208.92	208.92	0.64	0.4266

Figure 10: Pairwise Comparison of STPIS Changes in Day 2

		Day 2															
		Δ 5th vs.1st				Δ 6th vs.1st				Δ 7th vs.1st				Δ 8th vs.1st			
Contrast	DF	Contrast SS	Mean Square	F Value	Pr > F	Contrast SS	Mean Square	F Value	Pr > F	Contrast SS	Mean Square	F Value	Pr > F	Contrast SS	Mean Square	F Value	Pr > F
Compare Placebo & Treatment 3	1	5,291.43	5,291.43	17.78	<.0001	9,500.80	9,500.80	29.58	<.0001	12,781.85	12,781.85	34.52	<.0001	23,766.88	23,766.88	53.25	<.0001
Compare Placebo & Treatment 1	1	6,739.38	6,739.38	22.65	<.0001	9,419.92	9,419.92	29.32	<.0001	11,559.14	11,559.14	31.22	<.0001	23,058.42	23,058.42	51.66	<.0001
Compare Placebo & Treatment 2	1	6,113.48	6,113.48	20.54	<.0001	10,807.70	10,807.70	33.64	<.0001	11,416.47	11,416.47	30.83	<.0001	18,068.80	18,068.80	40.48	<.0001
Compare Treatment 3 & Treatment 1	1	46.98	46.98	0.16	0.6917	15.68	15.68	0.05	0.8255	94.68	94.68	0.26	0.6138	63.12	63.12	0.14	0.7074
Compare Treatment 3 & Treatment 2	1	10.85	10.85	0.04	0.8488	12.91	12.91	0.04	0.8414	95.54	95.54	0.26	0.6122	613.90	613.90	1.38	0.2427
Compare Treatment 1 & Treatment 2	1	13.40	13.40	0.05	0.8322	61.24	61.24	0.19	0.663	0.01	0.01	0	0.9958	307.61	307.61	0.69	0.4077

The line chart (Figure 11) illustrates the comparison of changes in STPIS means across the different intervention groups, with the X-axis representing time points (1st measure, 2nd measure, etc.), and the Y-axis representing the mean change in STPIS. These results suggest that Treatment 3 holds promise for effectively reducing the intensity of sore throat pain.

Figure 11: Changes in STPIS Means by Group

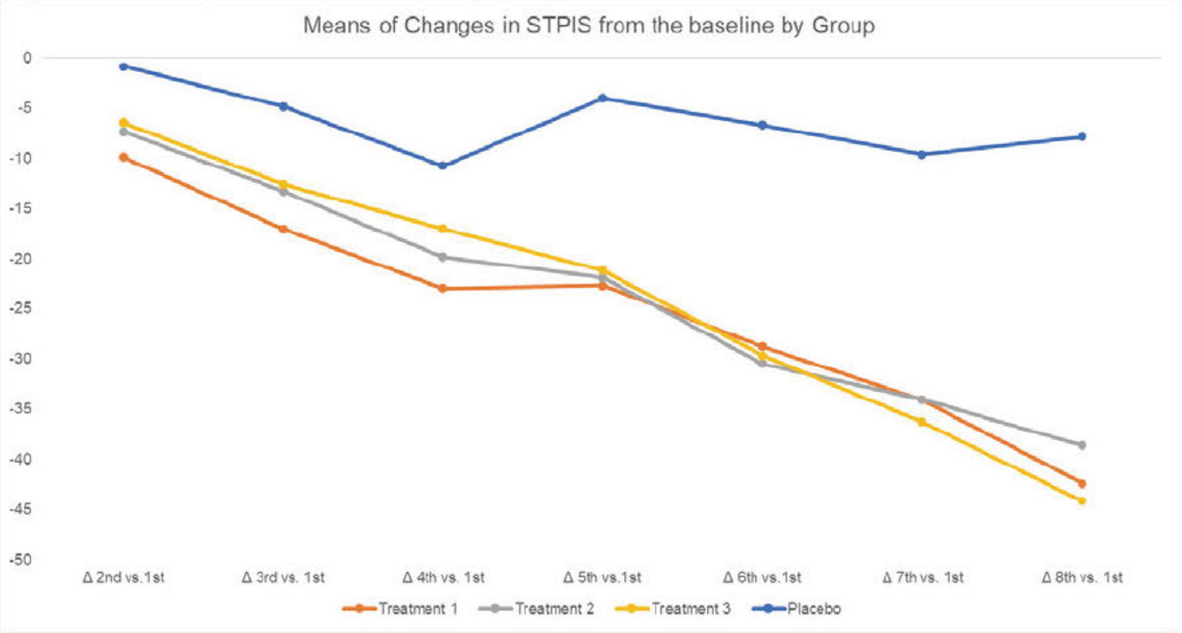

4.2.4. Repeated Measures Analysis of Jackson Scores

The objective of analyzing Jackson scores was to evaluate the effects of different drug groups on overall and individual cold symptoms over time. This section presents nine sets of Jackson score results: the modified Jackson score followed by eight individual Jackson symptoms assessments (nasal discharge–fever/chills). Each of the nine subsections that follow includes charts containing the following data:

- The repeated measures multivariate ANOVA test, which demonstrates whether there were statistically significant differences between the drug groups (where the p-value is less than 0.05).
- The significance test of the modified Jackson scores, as well as the changes in the modified Jackson scores from day 1 to day 2.
- The changes in mean modified Jackson scores across the different groups.
- The pairwise comparison analysis of the changes in the modified Jackson scores across the two days of the study.

Jackson Score: Modified Jackson Score

We performed a repeated measures analysis to examine within-subject changes in the modified Jackson scores across multiple time points (see Figure 12 and Figure 13), finding statistically significant differences among the groups at the overall level. No statistically significant differences among the intervention groups were observed in the modified Jackson scores on day 1 ($p > 0.05$), indicating similar symptom severity at baseline. However, significant differences were found among the intervention groups in the modified Jackson scores on day 2 ($p < 0.05$), suggesting divergent effects of the interventions on symptom severity at the follow-up assessment. Significant differences were also observed in the changes in the modified Jackson scores between day 1 and day 2 ($p < 0.05$), indicating varying treatment responses over the course of the study.

Figure 12: Multivariate ANOVA Test for the Modified Jackson Score

MANOVA Test Criteria and F Approximations for the Hypothesis of Modified Jackson Score * Group Effect				
Statistic	Value	F Value	Num DF	Pr>F
Wilks's Lambda	0.74	18.36	3	<.0001
Pillai's Trace	0.26	18.36	3	<.0001
Hotelling-Lawley Trace	0.36	18.36	3	<.0001
Roy's Greatest Root	0.36	18.36	3	<.0001

Figure 13: Significance Test and Day 1 vs. Day 2 Changes in the Modified Jackson Scores

	Day 1 Modified Jackson Score				Day 2 Modified Jackson Score				Δ Modified Jackson Score (Day 2 vs.Day 1)			
Group	Mean (95% CI)	SD	Median	P-Value	Mean (95% CI)	SD	Median	P-Value	Mean (95% CI)	SD	Median	P-Value
Placebo	7.06 (5.60 - 8.51)	4.24	6.00		7.26 (5.63 - 8.88)	4.74	8.00		0.20 (-0.83 - 1.23)	3.00	1.00	
Treatment 3	6.70 (5.38 - 8.03)	3.97	7.00	0.4243	2.11 (1.43 - 2.79)	2.04	2.00	<.0001	-4.59 (-5.62 - -3.57)	3.07	-4.00	<.0001
Treatment 1	5.88 (4.74 - 7.03)	3.73	4.00		3.63 (2.60 - 4.65)	3.33	3.00		-2.26 (-3.04 - -1.47)	2.56	-2.00	
Treatment 2	7.24 (5.95 - 8.53)	4.14	7.00		3.43 (2.58 - 4.28)	2.73	2.50		-3.81 (-4.82 - -2.80)	3.26	-3.50	

The bar chart in Figure 14 illustrates the changes in the modified Jackson mean scores between day 2 and the baseline score for each trial group (greater negative numbers mean greater improvement). The Treatment 3 group exhibited the largest mean change, indicating the most significant improvement in symptom severity compared to the other groups.

Figure 14: Mean of Changes in the Modified Jackson Scores Across the Different Groups

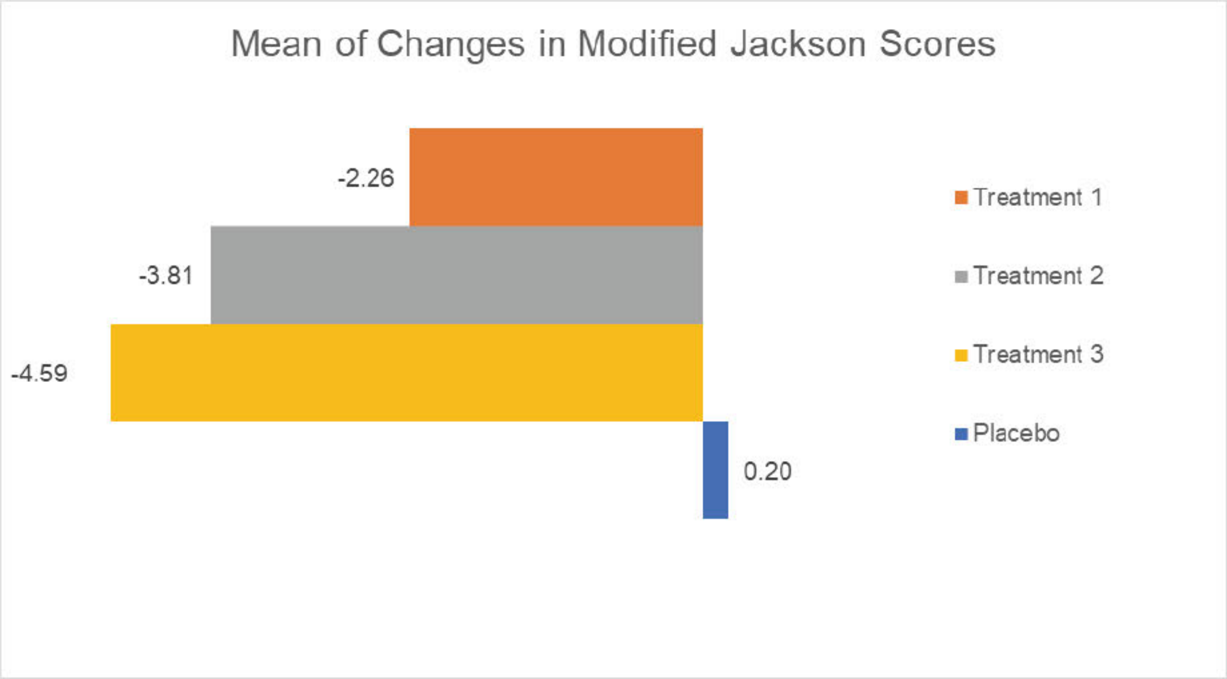

Post hoc pairwise comparisons revealed statistically significant differences between Treatment 3 and Treatment 1, as well as between Treatment 1 and Treatment 2 groups (see Figure 15). Strong statistical significance was also observed when comparing all Biovanta medicines to the placebo group, indicating a significant impact of the Biovanta interventions compared to the control group.

Figure 15: Pairwise Comparison Analysis of Changes in the Modified Jackson Scores

		Δ Modified Jackson Score (Day 2 vs. Day 1)			
Contrast	DF	Contrast SS	Mean Square	F Value	Pr > F
Compare Placebo & Treatment 3	1	413.47	413.47	46.75	<.0001
Compare Placebo & Treatment 1	1	116.37	116.37	13.16	0.0004
Compare Placebo & Treatment 2	1	306.91	306.91	34.7	<.0001
Compare Treatment 3 & Treatment 1	1	108.78	108.78	12.3	0.0006
Compare Treatment 3 & Treatment 2	1	12.12	12.12	1.37	0.2435
Compare Treatment 1 & Treatment 2	1	51.29	51.29	5.8	0.0172

Jackson Score: Nasal Congestion

The analysis of the nasal congestion symptom produced results very similar to the overall modified Jackson scores, so we are not restating them in this subsection. Please see the section **Jackson Score: Modified Jackson Score,** above, for detailed findings.

Notably, Treatment 3 demonstrated a stronger impact than the other groups (see Figure 16, Figure 17, Figure 18, and Figure 19).

Figure 16: Multivariate ANOVA Test for the Nasal Congestion Symptom

MANOVA Test Criteria and F Approximations for the Hypothesis of Nasal Congestion * Group Effect				
Statistic	Value	F Value	Num DF	Pr>F
Wilks's Lambda	0.87	7.7	3	<.0001
Pillai's Trace	0.13	7.7	3	<.0001
Hotelling-Lawley Trace	0.15	7.7	3	<.0001
Roy's Greatest Root	0.15	7.7	3	<.0001

Figure 17: Significance Test and Day 1 vs. Day 2 Changes in the Nasal Congestion Symptom

	Day 1 Nasal Congestion				Day 2 Nasal Congestion				Δ Nasal Congestion (Day 2 vs.Day 1)			
Group	Mean (95% CI)	SD	Median	P-Value	Mean (95% CI)	SD	Median	P-Value	Mean (95% CI)	SD	Median	P-Value
Placebo	0.91 (0.59 - 1.24)	0.95	1.00		0.86 (0.56 - 1.16)	0.88	1.00		-0.06 (-0.29 - 0.18)	0.68	0.00	
Treatment 3	1.08 (0.88 - 1.29)	0.60	1.00	0.4324	0.36 (0.20 - 0.53)	0.49	0.00	0.0135	-0.71 (-0.94 - -0.49)	0.67	-1.00	<.0001
Treatment 1	1.05 (0.80 - 1.29)	0.79	1.00		0.76 (0.51 - 1.02)	0.82	1.00		-0.26 (-0.51 - -0.01)	0.80	0.00	
Treatment 2	1.21 (0.96 - 1.47)	0.81	1.00		0.52 (0.34 - 0.71)	0.59	0.00		-0.69 (-0.90 - -0.48)	0.68	-1.00	

Figure 18: Mean of Changes in the Nasal Congestion Symptom Across the Different Groups

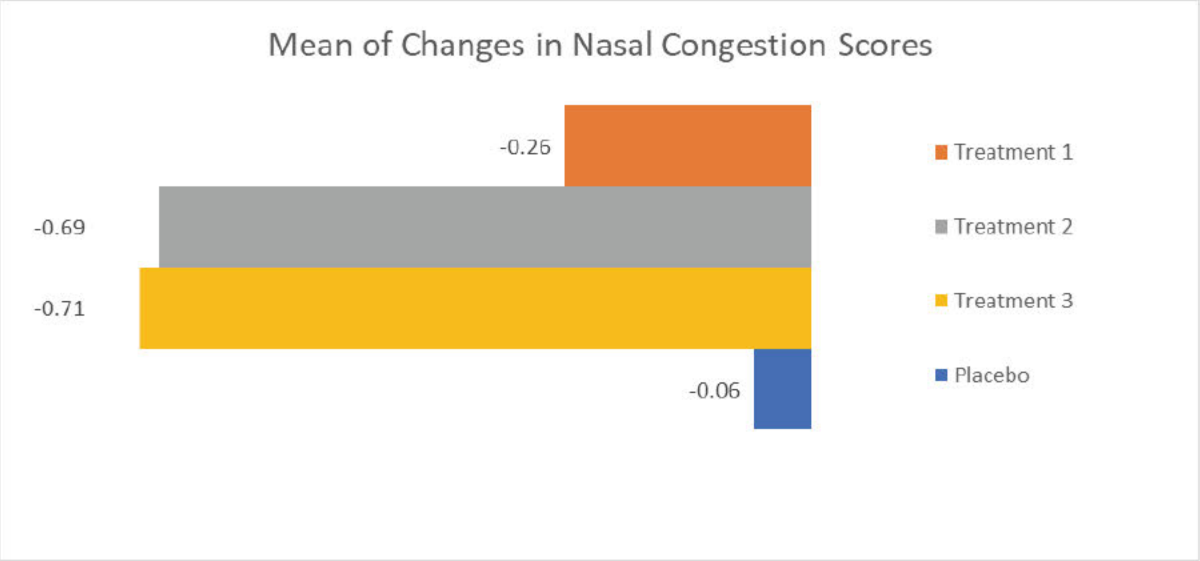

Figure 19: Pairwise Comparison Analysis of Changes in the Nasal Congestion Symptom

		Δ Nasal Congestion (Day 2 vs.Day 1)			
Contrast	DF	Contrast SS	Mean Square	F Value	Pr > F
Compare Placebo & Treatment 3	1	7.56	7.56	14.89	0.0002
Compare Placebo & Treatment 1	1	0.80	0.80	1.58	0.2111
Compare Placebo & Treatment 2	1	7.66	7.66	15.09	0.0002
Compare Treatment 3 & Treatment 1	1	3.91	3.91	7.7	0.0062
Compare Treatment 3 & Treatment 2	1	0.01	0.01	0.02	0.8841
Compare Treatment 1 & Treatment 2	1	3.86	3.86	7.6	0.0066

Jackson Score: Nasal Discharge

The analysis of the nasal discharge symptom produced results very similar to the overall modified Jackson scores, so we are not restating them in this subsection. Please see the section **Jackson Score: Modified Jackson Score**, above, for detailed findings.

The finding unique to the nasal discharge symptom was that Treatment 2 demonstrated a stronger impact than the other groups (see Figure 20, Figure 21, Figure 22, and Figure 23).

Figure 20: Multivariate ANOVA Test for the Nasal Discharge Symptom

MANOVA Test Criteria and F Approximations for the Hypothesis of Nasal Discharge * Group Effect				
Statistic	Value	F Value	Num DF	Pr>F
Wilks's Lambda	0.89	6.56	3	0.0003
Pillai's Trace	0.11	6.56	3	0.0003
Hotelling-Lawley Trace	0.13	6.56	3	0.0003
Roy's Greatest Root	0.13	6.56	3	0.0003

Figure 21: Significance Test and Day 1 vs. Day 2 Changes in the Nasal Discharge Symptom

	Day 1 Nasal Discharge				Day 2 Nasal Discharge				Δ Nasal Discharge (Day 2 vs.Day 1)			
Group	Mean (95% CI)	SD	Median	P-Value	Mean (95% CI)	SD	Median	P-Value	Mean (95% CI)	SD	Median	P-Value
Placebo	0.77 (0.44 - 1.11)	0.97	0.00		0.74 (0.42 - 1.07)	0.95	0.00		-0.03 (-0.21 - 0.15)	0.51	0.00	
Treatment 3	0.73 (0.45 - 1.01)	0.84	1.00	0.1086	0.30 (0.12 - 0.47)	0.52	0.00	0.0492	-0.43 (-0.68 - -0.19)	0.73	0.00	0.0003
Treatment 1	0.76 (0.55 - 0.98)	0.69	1.00		0.44 (0.25 - 0.64)	0.63	0.00		-0.31 (-0.51 - -0.11)	0.64	0.00	
Treatment 2	1.12 (0.88 - 1.36)	0.77	1.00		0.45 (0.27 - 0.64)	0.59	0.00		-0.67 (-0.87 - -0.46)	0.65	-1.00	

Figure 22: Mean of Changes in the Nasal Discharge Symptom Across the Different Groups

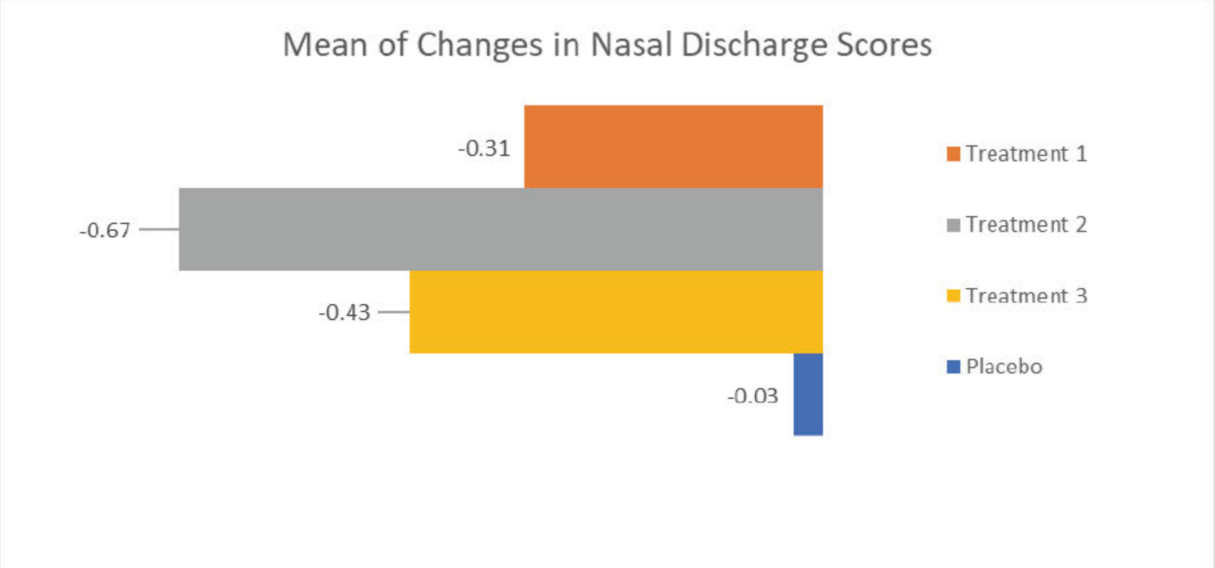

Figure 23: Pairwise Comparison Analysis of Changes in the Nasal Discharge Symptom

		Δ Nasal Discharge (Day 2 vs.Day 1)			
Contrast	DF	Contrast SS	Mean Square	F Value	Pr > F
Compare Placebo & Treatment 3	1	2.93	2.93	7.15	0.0083
Compare Placebo & Treatment 1	1	1.51	1.51	3.67	0.0572
Compare Placebo & Treatment 2	1	7.77	7.77	18.95	<.0001
Compare Treatment 3 & Treatment 1	1	0.30	0.30	0.72	0.3961
Compare Treatment 3 & Treatment 2	1	1.08	1.08	2.63	0.1069
Compare Treatment 1 & Treatment 2	1	2.68	2.68	6.53	0.0116

Jackson Score: Sneezing

The analysis of the sneezing symptom produced results very similar to the overall modified Jackson scores, so we are not restating them in this subsection. Please see the section **Jackson Score: Modified Jackson Score**, above, for detailed findings.

The finding unique to the sneezing symptom was that Treatment 2 demonstrated a stronger impact than the other groups (see Figure 24, Figure 25, Figure 26, and Figure 27).

Figure 24: Multivariate ANOVA Test for the Sneezing Symptom

MANOVA Test Criteria and F Approximations for the Hypothesis of Sneezing * Group Effect				
Statistic	Value	F Value	Num DF	Pr>F
Wilks's Lambda	0.92	4.32	3	0.0059
Pillai's Trace	0.08	4.32	3	0.0059
Hotelling-Lawley Trace	0.09	4.32	3	0.0059
Roy's Greatest Root	0.09	4.32	3	0.0059

Figure 25: Significance Test and Day 1 vs. Day 2 Changes in the Sneezing Symptom

	Day 1 Sneezing				Day 2 Sneezing				Δ Sneezing (Day 2 vs.Day 1)			
Group	Mean (95% CI)	SD	Median	P-Value	Mean (95% CI)	SD	Median	P-Value	Mean (95% CI)	SD	Median	P-Value
Placebo	0.89 (0.60 - 1.17)	0.83	1.00	0.0819	0.97 (0.66 - 1.29)	0.90	1.00	<.0001	0.09 (-0.13 - 0.30)	0.62	0.00	0.0059
Treatment 3	0.62 (0.37 - 0.87)	0.76	0.00		0.27 (0.10 - 0.44)	0.51	0.00		-0.35 (-0.56 - -0.14)	0.63	0.00	
Treatment 1	0.50 (0.29 - 0.71)	0.67	0.00		0.33 (0.14 - 0.52)	0.61	0.00		-0.12 (-0.31 - 0.07)	0.60	0.00	
Treatment 2	0.86 (0.59 - 1.12)	0.84	1.00		0.45 (0.27 - 0.64)	0.59	0.00		-0.40 (-0.64 - -0.17)	0.77	0.00	

Figure 26: Mean of Changes in the Sneezing Symptom Across the Different Groups

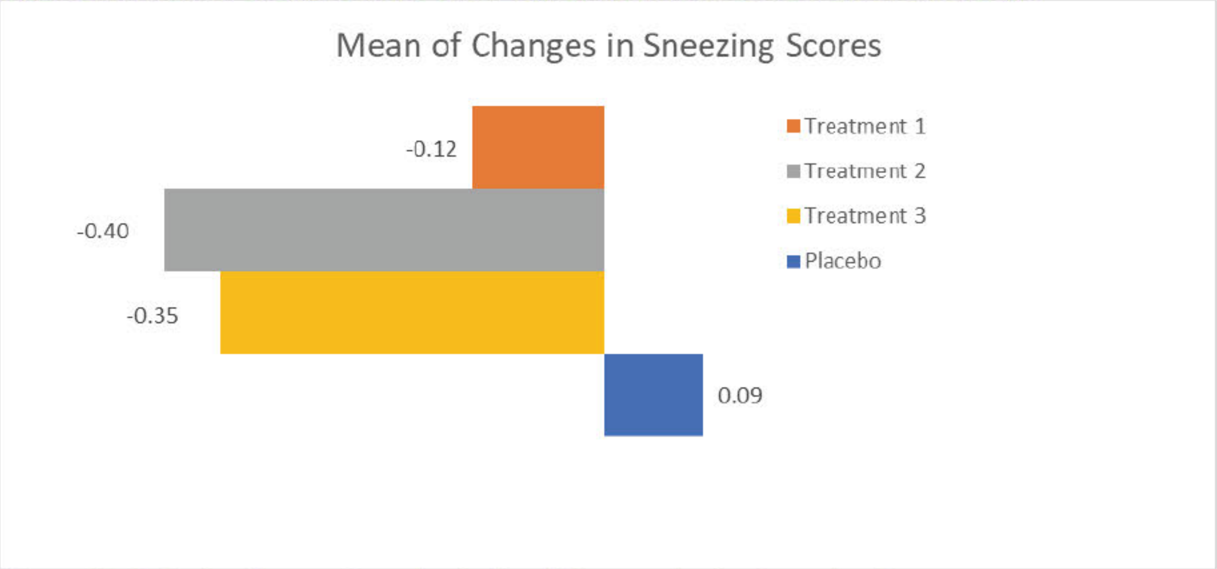

Figure 27: Pairwise Comparison Analysis of Changes in the Sneezing Symptom

		Δ Sneezing (Day 2 vs.Day 1)			
Contrast	DF	Contrast SS	Mean Square	F Value	Pr > F
Compare Placebo & Treatment 3	1	3.42	3.42	7.82	0.0058
Compare Placebo & Treatment 1	1	0.82	0.82	1.88	0.1729
Compare Placebo & Treatment 2	1	4.57	4.57	10.43	0.0015
Compare Treatment 3 & Treatment 1	1	1.02	1.02	2.34	0.1284
Compare Treatment 3 & Treatment 2	1	0.06	0.06	0.13	0.7208
Compare Treatment 1 & Treatment 2	1	1.66	1.66	3.79	0.0534

Jackson Score: Sore Throat

The analysis of the sore throat symptom produced results very similar to the overall modified Jackson scores, so we are not restating them in this subsection. Please see the section **Jackson Score: Modified Jackson Score**, above, for detailed findings.

Notably, Treatment 3 demonstrated a stronger impact than the other groups (see Figure 28, Figure 29, Figure 30, and Figure 31).

Figure 28: Multivariate ANOVA Test for the Sore Throat Symptom

MANOVA Test Criteria and F Approximations for the Hypothesis of Sore Throat * Group Effect				
Statistic	Value	F Value	Num DF	Pr>F
Wilks's Lambda	0.88	6.75	3	0.0003
Pillai's Trace	0.12	6.75	3	0.0003
Hotelling-Lawley Trace	0.13	6.75	3	0.0003
Roy's Greatest Root	0.13	6.75	3	0.0003

Figure 29: Significance Test and Day 1 vs. Day 2 Changes in the Sore Throat Symptom

	Day 1 Sore Throat				Day 2 Sore Throat				Δ Sore Throat (Day 2 vs.Day 1)			
Group	Mean (95% CI)	SD	Median	P-Value	Mean (95% CI)	SD	Median	P-Value	Mean (95% CI)	SD	Median	P-Value
Placebo	1.71 (1.46 - 1.96)	0.72	2.00		1.41 (1.08 - 1.75)	0.96	2.00		-0.29 (-0.53 - -0.06)	0.68	0.00	
Treatment 3	1.59 (1.34 - 1.85)	0.76	2.00	0.5839	0.54 (0.35 - 0.73)	0.56	1.00	<.0001	-1.05 (-1.30 - -0.81)	0.74	-1.00	0.0003
Treatment 1	1.60 (1.38 - 1.83)	0.73	2.00		0.88 (0.66 - 1.11)	0.73	1.00		-0.72 (-0.96 - -0.49)	0.77	-1.00	
Treatment 2	1.48 (1.27 - 1.69)	0.67	2.00		0.69 (0.50 - 0.88)	0.60	1.00		-0.79 (-1.00 - -0.57)	0.68	-1.00	

Figure 30: Mean of Changes in the Sore Throat Symptom Across the Different Groups

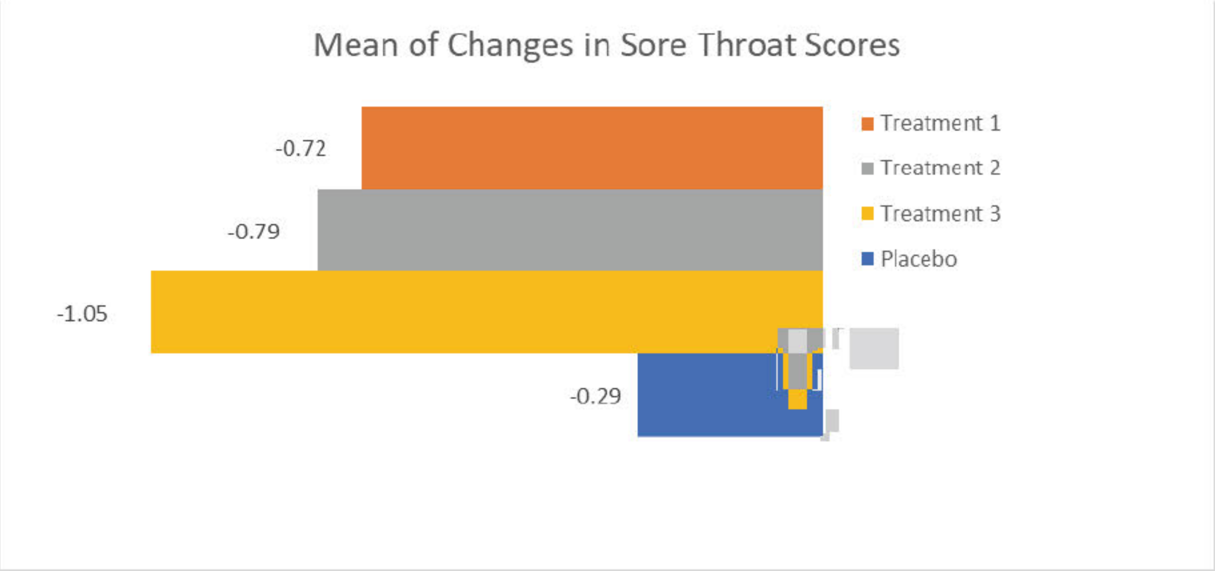

Figure 31: Pairwise Comparison Analysis of Changes in the Sore Throat Symptom

Contrast	DF	Δ Sore Throat (Day 2 vs.Day 1) Contrast SS	Mean Square	F Value	Pr > F
Compare Placebo & Treatment 3	1	10.23	10.23	19.77	<.0001
Compare Placebo & Treatment 1	1	3.46	3.46	6.68	0.0107
Compare Placebo & Treatment 2	1	4.54	4.54	8.77	0.0035
Compare Treatment 3 & Treatment 1	1	2.21	2.21	4.26	0.0406
Compare Treatment 3 & Treatment 2	1	1.42	1.42	2.74	0.1001
Compare Treatment 1 & Treatment 2	1	0.09	0.09	0.17	0.6787

Jackson Score: Cough

The analysis of the cough symptom produced results very similar to the overall modified Jackson scores, so we are not restating them in this subsection. Please see the section **Jackson Score: Modified Jackson Score**, above, for detailed findings.

Notably, Treatment 3 demonstrated a stronger impact than the other groups (see Figure 32, Figure 33, Figure 34, and Figure 35).

Figure 32: Multivariate ANOVA Test for the Cough Symptom

MANOVA Test Criteria and F Approximations for the Hypothesis of Cough * Group Effect				
Statistic	Value	F Value	Num DF	Pr>F
Wilks's Lambda	0.86	7.85	3	<.0001
Pillai's Trace	0.14	7.85	3	<.0001
Hotelling-Lawley Trace	0.16	7.85	3	<.0001
Roy's Greatest Root	0.16	7.85	3	<.0001

Figure 33: Significance Test and Day 1 vs. Day 2 Changes in the Cough Symptom

	Day 1 Cough				Day 2 Cough				Δ Cough (Day 2 vs.Day 1)			
Group	Mean (95% CI)	SD	Median	P-Value	Mean (95% CI)	SD	Median	P-Value	Mean (95% CI)	SD	Median	P-Value
Placebo	0.94 (0.64 - 1.24)	0.85	1.00		1.06 (0.74 - 1.38)	0.90	1.00		0.12 (-0.13 - 0.37)	0.70	0.00	
Treatment 3	0.95 (0.64 - 1.25)	0.91	1.00	0.2958	0.27 (0.10 - 0.44)	0.51	0.00	<.0001	-0.68 (-0.92 - -0.43)	0.75	-1.00	<.0001
Treatment 1	0.64 (0.39 - 0.90)	0.82	0.00		0.49 (0.25 - 0.72)	0.77	0.00		-0.17 (-0.40 - 0.07)	0.76	0.00	
Treatment 2	0.93 (0.68 - 1.18)	0.81	1.00		0.50 (0.31 - 0.69)	0.59	0.00		-0.43 (-0.65 - -0.21)	0.70	0.00	

Figure 34: Mean of Changes in the Cough Symptom Across the Different Groups

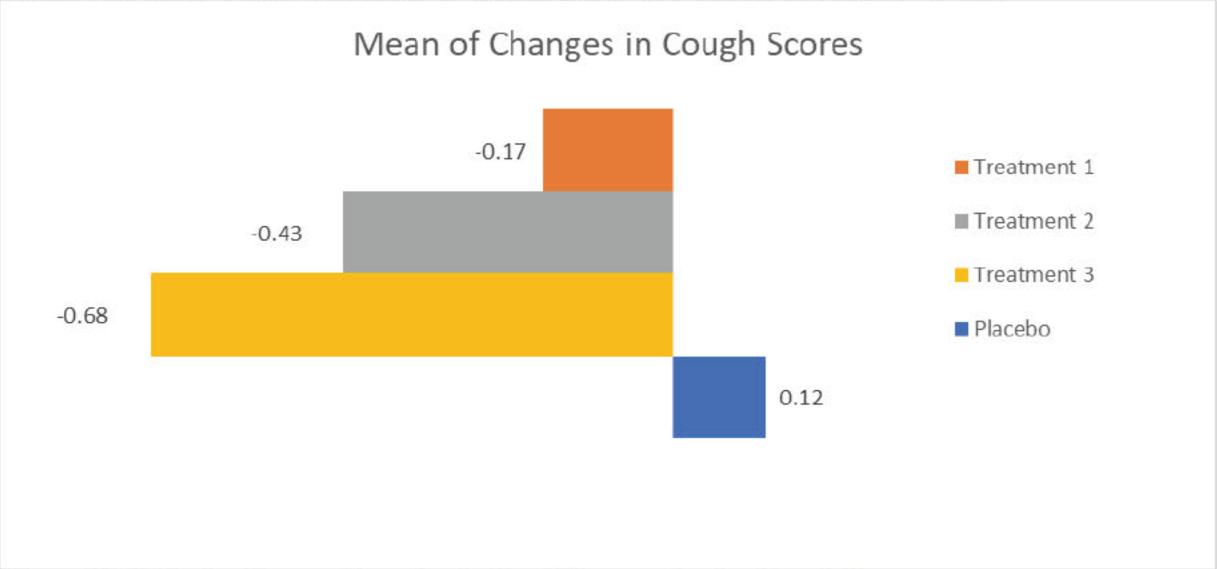

Figure 35: Pairwise Comparison Analysis of Changes in the Cough Symptom

		Δ Cough (Day 2 vs.Day 1)			
Contrast	DF	Contrast SS	Mean Square	F Value	Pr > F
Compare Placebo & Treatment 3	1	11.08	11.08	20.84	<.0001
Compare Placebo & Treatment 1	1	1.53	1.53	2.88	0.0917
Compare Placebo & Treatment 2	1	5.59	5.59	10.51	0.0015
Compare Treatment 3 & Treatment 1	1	5.10	5.10	9.59	0.0023
Compare Treatment 3 & Treatment 2	1	1.20	1.20	2.26	0.1349
Compare Treatment 1 & Treatment 2	1	1.44	1.44	2.71	0.1018

Jackson Score: Headache

The analysis of the headache symptom produced results very similar to the overall modified Jackson scores, so we are not restating them in this subsection. Please see the section **Jackson Score: Modified Jackson Score**, above, for detailed findings.

Notably, Treatment 3 demonstrated a stronger impact than the other groups (see Figure 36, Figure 37, Figure 38, and Figure 39).

Figure 36: Multivariate ANOVA Test for the Headache Symptom

MANOVA Test Criteria and F Approximations for the Hypothesis of Headache * Group Effect				
Statistic	Value	F Value	Num DF	Pr>F
Wilks's Lambda	0.83	9.98	3	<.0001
Pillai's Trace	0.17	9.98	3	<.0001
Hotelling-Lawley Trace	0.20	9.98	3	<.0001
Roy's Greatest Root	0.20	9.98	3	<.0001

Figure 37: Significance Test and Day 1 vs. Day 2 Changes in the Headache Symptom

	Day 1 Headache				Day 2 Headache				Δ Headache (Day 2 vs.Day 1)			
Group	Mean (95% CI)	SD	Median	P-Value	Mean (95% CI)	SD	Median	P-Value	Mean (95% CI)	SD	Median	P-Value
Placebo	0.70 (0.42 - 0.97)	0.77	1.00		0.91 (0.60 - 1.23)	0.92	1.00		0.27 (0.02 - 0.53)	0.72	0.00	
Treatment 3	0.62 (0.37 - 0.87)	0.76	0.00	0.5127	0.14 (0.02 - 0.25)	0.35	0.00	<.0001	-0.49 (-0.72 - -0.26)	0.69	0.00	<.0001
Treatment 1	0.47 (0.25 - 0.68)	0.70	0.00		0.26 (0.10 - 0.41)	0.49	0.00		-0.21 (-0.35 - -0.07)	0.47	0.00	
Treatment 2	0.50 (0.26 - 0.74)	0.77	0.00		0.26 (0.11 - 0.42)	0.50	0.00		-0.24 (-0.39 - -0.09)	0.48	0.00	

Figure 38: Mean of Changes in the Headache Symptom Across the Different Groups

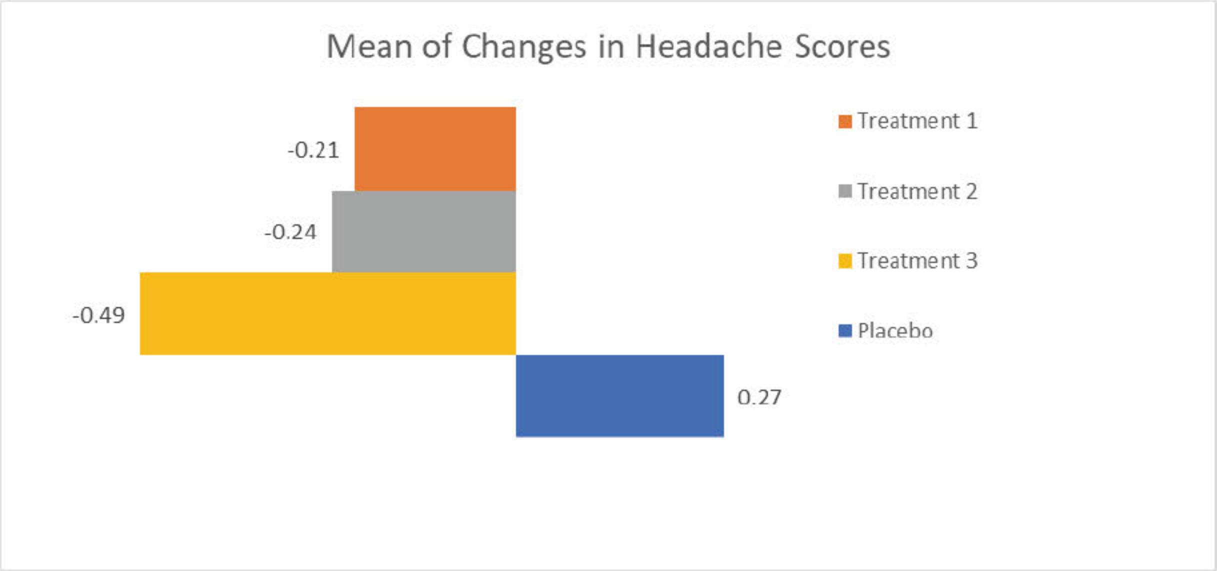

Figure 39: Pairwise Comparison Analysis of Changes in the Headache Symptom

		Δ Headache (Day 2 vs.Day 1)			
Contrast	DF	Contrast SS	Mean Square	F Value	Pr > F
Compare Placebo & Treatment 3	1	10.05	10.05	28.9	<.0001
Compare Placebo & Treatment 1	1	4.34	4.34	12.47	0.0005
Compare Placebo & Treatment 2	1	4.82	4.82	13.86	0.0003
Compare Treatment 3 & Treatment 1	1	1.53	1.53	4.39	0.0378
Compare Treatment 3 & Treatment 2	1	1.21	1.21	3.49	0.0637
Compare Treatment 1 & Treatment 2	1	0.02	0.02	0.05	0.8223

Jackson Score: Malaise

The analysis of the malaise symptom produced results very similar to the overall modified Jackson scores, so we are not restating them in this subsection. Please see the section **Jackson Score: Modified Jackson Score**, above, for detailed findings.

Notably, Treatment 3 demonstrated a stronger impact than the other groups (see Figure 40, Figure 41, Figure 42, and Figure 43).

Figure 40: Multivariate ANOVA Test for the Malaise Symptom

MANOVA Test Criteria and F Approximations for the Hypothesis of Malaise * Group Effect				
Statistic	Value	F Value	Num DF	Pr>F
Wilks's Lambda	0.89	6.45	3	0.0004
Pillai's Trace	0.11	6.45	3	0.0004
Hotelling-Lawley Trace	0.13	6.45	3	0.0004
Roy's Greatest Root	0.13	6.45	3	0.0004

Figure 41: Significance Test and Day 1 vs. Day 2 Changes in the Malaise Symptom

	Day 1 Malaise				Day 2 Malaise				Δ Malasie (Day 2 vs.Day 1)			
Group	Mean (95% CI)	SD	Median	P-Value	Mean (95% CI)	SD	Median	P-Value	Mean (95% CI)	SD	Median	P-Value
Placebo	0.94 (0.65 - 1.23)	0.84	1.00		1.00 (0.68 - 1.32)	0.94	1.00		0.06 (-0.18 - 0.29)	0.68	0.00	
Treatment 3	0.84 (0.58 - 1.09)	0.76	1.00	0.4518	0.19 (0.04 - 0.34)	0.46	0.00	<.0001	-0.65 (-0.90 - -0.40)	0.75	-1.00	0.0004
Treatment 1	0.64 (0.37 - 0.92)	0.88	0.00		0.33 (0.13 - 0.52)	0.64	0.00		-0.33 (-0.51 - -0.16)	0.57	0.00	
Treatment 2	0.80 (0.55 - 1.06)	0.81	1.00		0.40 (0.25 - 0.56)	0.50	0.00		-0.39 (-0.62 - -0.16)	0.74	0.00	

Figure 42: Mean of Changes in the Malaise Symptom Across the Different Groups

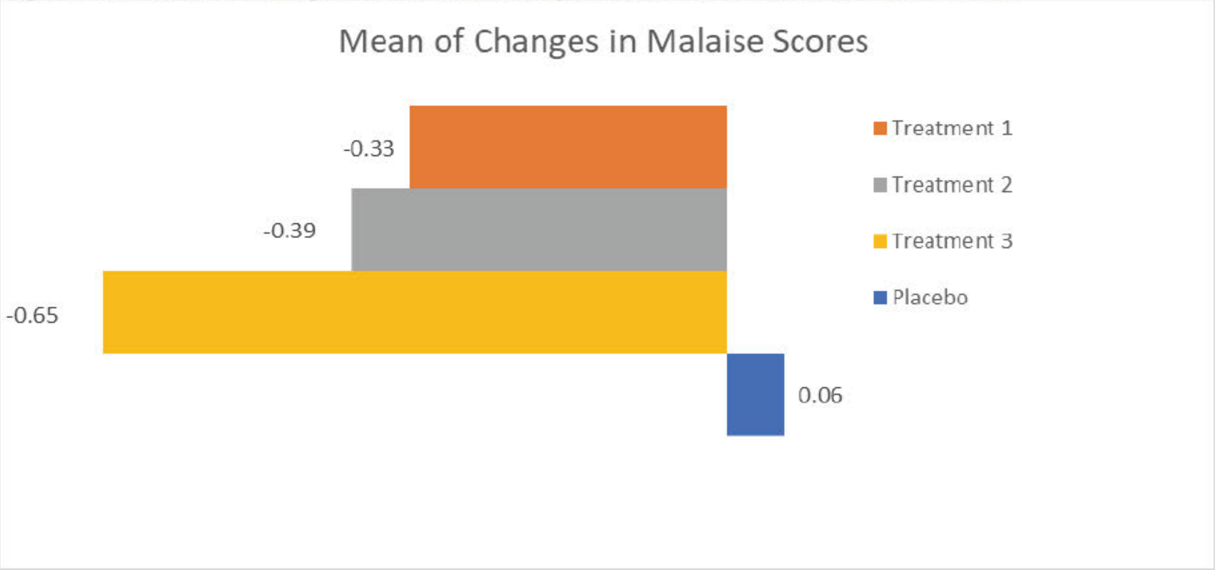

Figure 43: Pairwise Comparison Analysis of Changes in the Malaise Symptom

		Δ Malasie (Day 2 vs.Day 1)			
Contrast	DF	Contrast SS	Mean Square	F Value	Pr > F
Compare Placebo & Treatment 3	1	8.96	8.96	18.95	<.0001
Compare Placebo & Treatment 1	1	2.91	2.91	6.16	0.0142
Compare Placebo & Treatment 2	1	3.78	3.78	7.99	0.0053
Compare Treatment 3 & Treatment 1	1	1.96	1.96	4.14	0.0437
Compare Treatment 3 & Treatment 2	1	1.30	1.30	2.75	0.0996
Compare Treatment 1 & Treatment 2	1	0.07	0.07	0.14	0.7067

Jackson Score: Fever/Chills

The analysis of the fever/chills symptom produced results very similar to the overall modified Jackson scores, so we are not restating them in this subsection. Please see the section **Jackson Score: Modified Jackson Score**, above, for detailed findings.

Notably, Treatment 3 demonstrated a stronger impact than the other groups (see Figure 44, Figure 45, Figure 46, and Figure 47).

Figure 44: Multivariate ANOVA Test for the Fever/Chills Symptom

MANOVA Test Criteria and F Approximations for the Hypothesis of Fever/Chills * Group Effect				
Statistic	Value	F Value	Num DF	Pr>F
Wilks's Lambda	0.92	4.58	3	0.0042
Pillai's Trace	0.08	4.58	3	0.0042
Hotelling-Lawley Trace	0.09	4.58	3	0.0042
Roy's Greatest Root	0.09	4.58	3	0.0042

Figure 45: Significance Test and Day 1 vs. Day 2 Changes in the Fever/Chills Symptom

	Day 1 Fever/Chills				Day 2 Fever/Chills				Δ Fever/Chills (Day 2 vs.Day 1)			
Group	Mean (95% CI)	SD	Median	P-Value	Mean (95% CI)	SD	Median	P-Value	Mean (95% CI)	SD	Median	P-Value
Placebo	0.31 (0.13 - 0.50)	0.53	0.00		0.43 (0.22 - 0.64)	0.61	0.00		0.11 (-0.02 - 0.25)	0.40	0.00	
Treatment 3	0.30 (0.11 - 0.49)	0.57	0.00	0.9399	0.05 (-0.02 - 0.13)	0.23	0.00	0.0019	-0.24 (-0.41 - -0.08)	0.49	0.00	0.0042
Treatment 1	0.28 (0.10 - 0.46)	0.59	0.00		0.16 (0.03 - 0.30)	0.43	0.00		-0.12 (-0.22 - -0.02)	0.32	0.00	
Treatment 2	0.36 (0.15 - 0.56)	0.66	0.00		0.14 (0.03 - 0.25)	0.35	0.00		-0.21 (-0.39 - -0.04)	0.56	0.00	

Figure 46: Mean of Changes in the Fever/Chills Symptom Across the Different Groups

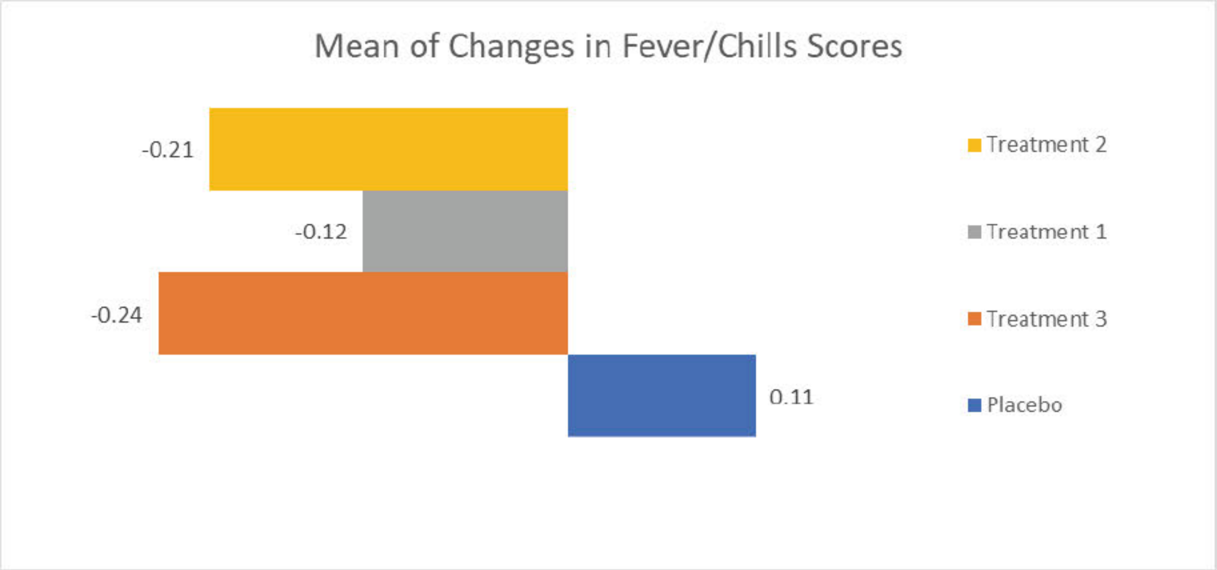

Figure 47: Pairwise Comparison Analysis of Changes in the Fever/Chills Symptom

		Δ Fever/Chills (Day 2 vs.Day 1)			
Contrast	DF	Contrast SS	Mean Square	F Value	Pr > F
Compare Placebo & Treatment 3	1	2.30	2.30	11.05	0.0011
Compare Placebo & Treatment 1	1	1.03	1.03	4.93	0.0279
Compare Placebo & Treatment 2	1	2.06	2.06	9.9	0.002
Compare Treatment 3 & Treatment 1	1	0.32	0.32	1.54	0.2165
Compare Treatment 3 & Treatment 2	1	0.02	0.02	0.08	0.7787
Compare Treatment 1 & Treatment 2	1	0.20	0.20	0.98	0.3236

4.2.5. Analysis of “Do you still have a cold?” Responses

The objective of analyzing the “Do you still have a cold?” variable was to examine changes in participants’ perception of feeling sick over time. A chi-square analysis revealed no statistically significant differences in participants’ responses to the question “Do you still have a cold?” across the two days of study.

Figure 48: Chi-square Frequency of "Do you still have a cold?" – Day 1

	Day 1			
Group	No	Yes	Total	P-Value
Placebo	18 (11.54%)	17 (10.90%)	35	
Treatment 3	11 (7.05%)	25 (16.03%)	36	
Treatment 1	19 (12.18%)	24 (15.38%)	43	0.0814
Treatment 2	11 (7.05%)	31 (19.87%)	42	
**Total**	**59 (37.82%)**	**97 (62.18%)**	**156**	

Figure 49: Chi-square Frequency of "Do you still have a cold?" – Day 2

	Day 2			
Group	No	Yes	Total	P-Value
Placebo	17 (11.04%)	16 (10.39%)	33	
Treatment 3	24 (15.58%)	12 (7.79%)	36	
Treatment 1	31 (20.13%)	12 (7.79%)	43	0.0837
Treatment 2	33 (21.43%)	9 (5.84%)	42	
**Total**	**105 (68.18%)**	**49 (31.82%)**	**154**	

Fisher's exact test analysis was performed on the re-coded "Do you still have a cold?" variable to examine the differences among the participant groups. The analysis detected a statistically significant difference among the groups (p-value < 0.05). The placebo group performed the worst, demonstrating either no impact at all or negative impact. All of the Biovanta products showed positive impact, though it is not appropriate to try to further statistically differentiate the impact among the Biovanta groups, because the "Do you still have a cold?" variable is categorical and unbalanced.

Figure 50: Fisher's Test Frequency Table

	Recovery from the cold : Compared with Day 1					
Group	Better	No Cold	The Same	Worse	Total	P-Value
Placebo	2 (1.31%)	15 (9.80%)	14 (9.15%)	2 (1.31%)	33	
Treatment 3	12 (7.84%)	11 (7.19%)	12 (7.84%)	0 (0.00%)	35	
Treatment 1	12 (7.84%)	19 (12.42%)	12 (7.84%)	0 (0.00%)	43	<.0001
Treatment 2	22 (14.38%)	11 (7.19%)	9 (5.88%)	0 (0.00%)	42	
**Total**	**48 (31.37%)**	**56 (36.60%)**	**47 (30.72%)**	**2 (1.31%)**	**153**	
