# Supplementary material for: Supporting respiratory epithelia and lowering inflammation to effectively treat common cold symptoms: A randomized controlled trial
Source: PLoS One. 2024 Nov 27;19(11):e0301959. doi: 10.1371/journal.pone.0301959 (PMC11602100; doi:10.1371/journal.pone.0301959)
Supplement: S3 File — (PDF) [file pone.0301959.s003.pdf]

Clinical Trial Experimental Groups and Placebo

Treatment 1: Biovanta (plus placebo pill)

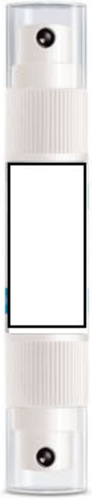

Step 1: Biovanta Aqueous Solution

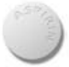

Placebo Pill

Step 2: Biovanta Organic Solution

Treatment 2: Biovanta wintergreen (plus placebo pill)

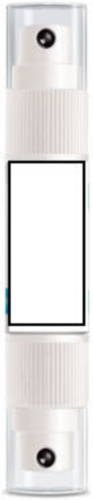

Step 1: Biovanta Aqueous Solution

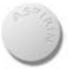

Placebo Pill

Step 2: Biovanta Organic Solution – wintergreen oil instead of menthol

Treatment 3: Biovanta wintergreen (plus aspirin)

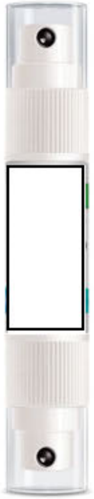

Step 1: Biovanta
Aqueous Solution containing lysozyme and lactoferrin

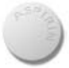

325 mg Aspirin

Step 2: Biovanta
Organic Solution wintergreen oil instead of menthol

Placebo

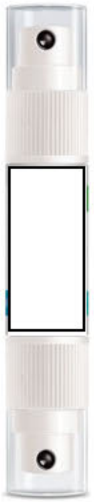

Step 1: Aqueous Solution

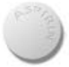

Placebo Pill

Step 2: Organic Solution

Participant Code:

Sore Throat Study

Patient Form

Title: **Alleviation of Acute Sore Throat Pain and other cold symptoms**

Primary Investigator:

Dr. [redacted]
Email: [redacted]
Phone: [redacted]

Adverse Events/Urgent Medical Questions:

Dr. [redacted]
Email: [redacted]
Phone: [redacted]

General Questions:

Email: [redacted]
Phone: [redacted]

Patient Forms/Instructions

1. Do not take any over the counter medications while enrolled in the study.
2. Please start the treatments the morning after your virtual visit with the physician as soon as you wake up.
3. When you wake up fill out the form, have something to eat, drink a glass of water, take a tablet and then administer one spray from each head of the spray bottle deep into your throat.
4. Repeat the spray administration every hour. Take one pill every 4 hours.
5. Continue this schedule during waking hours.
6. Complete the Forms for sore throat and cold symptoms four times a day: Once right before you take your first pill and sprays at the start of the day, and then once every four hours for a total of 4 surveys a day. Do the survey before you use the spray. Try your best to take the medication and fill out the forms at the same time every day.
7. If your symptoms get much worse during the course of the study, if you develop a fever, if you develop extreme difficulty swallowing, or if you feel you have any adverse events please contact [redacted], our Adverse Event Specialist via phone at [redacted] or by email at [redacted] Please feel free to contact him at any time for any reason.

DAY 1

The following chart will help you to remember when to take each dosage and when you need to take the **Sore Throat Assessments** and the **Jackson Score Assessment** further below. **Check off the times after completed. Hour 0 is the time you wake up.**

Day 1 Wake time: ________

Check off	Hour	Action
☐<br>☐<br><br>☐	0(a)<br>0(b)<br><br>0(c)	Mark "First Entry" on the DAY 1 "Sore Throat Assessment"<br>Take a single pill (have something to eat and a glass of water before you do so)<br>Administer a single puff from each end of the spray bottle
☐	1	Administer a single puff from each end of spray bottle (don't eat or drink for 5 min after).
☐	2	Administer a single puff from each end of spray bottle (don't eat or drink for 5 min after).
☐	3	Administer a single puff from each end of spray bottle (don't eat or drink for 5 min after).
☐<br>☐<br><br>☐	4(a)<br>4(b)<br><br>4(c)	Mark "Second Entry" on the DAY 1 "Sore Throat Assessment"<br>Take a single pill (have something to eat and a glass of water before you do so)<br>Administer a single puff from each end of spray bottle
☐	5	Administer a single puff from each end of spray bottle (don't eat or drink for 5 min after)
☐	6	Administer a single puff from each end of spray bottle (don't eat or drink for 5 min after)
☐	7	Administer a single puff from each end of spray bottle (don't eat or drink for 5 min after)

☐<br>☐<br><br>☐	8(a)<br>8(b)<br><br>8(c)	Mark “Third Entry” on the DAY 1 “Sore Throat Assessment”<br>Take a single pill (have something to eat and a glass of water before)<br>Administer a single puff from each end of spray bottle
☐	9	Administer a single puff from each end of spray bottle (don’t eat or drink for 5 min after)
☐	10	Administer a single puff from each end of spray bottle (don’t eat or drink for 5 min after)
☐	11	Administer a single puff from each end of spray bottle (don’t eat or drink for 5 min after)
☐<br>☐<br><br>☐	12 (a)<br>12 (b)<br><br>12 (c)	Mark “Fourth Entry” on the DAY 1 “Sore Throat Assessment”<br>Take a single pill (have something to eat and a glass of water before)<br>Administer a single puff from each end of spray bottle

DAY 1

Sore Throat Assessment:

Swallow and then place a line on the scale below indicating your level of sore throat pain (Sore Throat Pain Intensity Scale, STPIS)

First Entry

NO PAIN ________________________________ SEVERE PAIN

Second Entry

NO PAIN ________________________________ SEVERE PAIN

Third Entry

NO PAIN ________________________________ SEVERE PAIN

Fourth Entry

NO PAIN ________________________________ SEVERE PAIN

Day 1

Jackson Score Assessment:

Day 1: Modified Jackson Score

	Absent	Mild	Moderate	Severe
Nasal Congestion(blocked/stuffy nose)	○	○	○	○
Nasal Discharge(runny nose)	○	○	○	○
Sneezing	○	○	○	○
Sore/Scratchy Throat	○	○	○	○
Cough	○	○	○	○
Headache	○	○	○	○
Malaise(feeling generally unwell)	○	○	○	○
Fever/Chills(feeling hot/cold and shivery)	○	○	○	○

Day 1:
Do you still have a cold or feel like you're coming down with a cold?

○ Yes
○ No

DAY 2

The following chart will help you to remember when to take each dosage and when you need to take **Sore Throat Assessments** and the **Jackson Score Assessment** further below. **Check off the boxes after completed.**

Day 2 Wake time: ________

Check off	Hour	Action
☐<br>☐<br><br>☐	0(a)<br>0(b)<br><br>0(c)	Mark "First Entry" on the DAY 2 "Sore Throat Assessment"<br>Take a single pill (have something to eat and a glass of water before you do so)<br>Administer a single puff from each end of the spray bottle
☐	1	Administer a single puff from each end of spray bottle (don't eat or drink for 5 min after).
☐	2	Administer a single puff from each end of spray bottle (don't eat or drink for 5 min after).
☐	3	Administer a single puff from each end of spray bottle (don't eat or drink for 5 min after).
☐<br>☐<br><br>☐	4(a)<br>4(b)<br><br>4(c)	Mark "Second Entry" on the DAY 2 "Sore Throat Assessment"<br>Take a single pill (have something to eat and a glass of water before you do so)<br>Administer a single puff from each end of spray bottle
☐	5	Administer a single puff from each end of spray bottle (don't eat or drink for 5 min after)
☐	6	Administer a single puff from each end of spray bottle (don't eat or drink for 5 min after)
☐	7	Administer a single puff from each end of spray bottle (don't eat or drink for 5 min after)

☐<br>☐<br><br>☐	8(a)<br>8(b)<br><br>8(c)	Mark "Third Entry" on the DAY 2 "Sore Throat Assessment"<br>Take a single pill (have something to eat and a glass of water before)<br>Administer a single puff from each end of spray bottle
☐	9	Administer a single puff from each end of spray bottle (don't eat or drink for 5 min after)
☐	10	Administer a single puff from each end of spray bottle (don't eat or drink for 5 min after)
☐	11	Administer a single puff from each end of spray bottle (don't eat or drink for 5 min after)
☐<br>☐<br><br>☐	12 (a)<br>12 (b)<br><br>12 (c)	Mark "Fourth Entry" on the DAY 2 "Sore Throat Assessment"<br>Take a single pill (have something to eat and a glass of water before)<br>Administer a single puff from each end of spray bottle

DAY 2

Sore Throat Assessment:

Swallow and then place a line on the scale below indicating your level of sore throat pain (Sore Throat Pain Intensity Scale, STPIS)

First Entry

NO PAIN ______________________________ SEVERE PAIN

Second Entry

NO PAIN ______________________________ SEVERE PAIN

Third Entry

NO PAIN ______________________________ SEVERE PAIN

Fourth Entry

NO PAIN ______________________________ SEVERE PAIN

Day 2

Jackson Score Assessment:

Day 2: Modified Jackson Score

	Absent	Mild	Moderate	Severe
Nasal Congestion(blocked/stuffy nose)	○	○	○	○
Nasal Discharge(runny nose)	○	○	○	○
Sneezing	○	○	○	○
Sore/Scratchy Throat	○	○	○	○
Cough	○	○	○	○
Headache	○	○	○	○
Malaise(feeling generally unwell)	○	○	○	○
Fever/Chills(feeling hot/cold and shivery)	○	○	○	○

Day 2:
Do you still have a cold or feel like you're coming down with a cold?

○ Yes
○ No
